# Supplementary material for: Guest-host doped strategy for constructing ultralong-lifetime near-infrared organic phosphorescence materials for bioimaging
Source: Nat Commun. 2022 Jan 10;13:186. doi: 10.1038/s41467-021-27914-0 (PMC8748955; doi:10.1038/s41467-021-27914-0)
Supplement: Supplementary file 1 — Supplementary Information [file 41467_2021_27914_MOESM1_ESM.pdf]

Supplementary Information

Guest-Host doped Strategy for Constructing Ultralong-lifetime Near-infrared Organic Phosphorescence Materials for Bioimaging

Fuming Xiao‡[a], Heqi Gao‡[b], Yunxiang Lei*[a], Wenbo Dai[c], Miaochang Liu[a], Xiaoyan Zheng[c], Zhengxu Cai[c], Xiaobo Huang*[a], Huayue Wu[a] and Dan Ding*[b]

[a] School of Chemistry and Materials Engineering, Wenzhou University. Wenzhou 325035 (China)

[b] State Key Laboratory of Medicinal Chemical Biology, Key Laboratory of Bioactive Materials, Ministry of Education, and College of Life Sciences, Nankai University, Tianjin 300071 (China)

[c] School of Materials Science & Engineering, Beijing Institute of Technology, Beijing, 10081 (China)

E-mail: yunxianglei@wzu.edu.cn (Y. Lei); xiaobhuang@wzu.edu.cn (X. Huang); dingd@nankai.edu.cn (D. Ding).

‡ These authors contributed equally to this work.

Supplementary Figures

1. Molecular structure of organic RTP materials

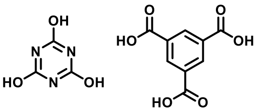
406 nm, 1560 ms
Nat. Commun. 2020, **11**, 4802.

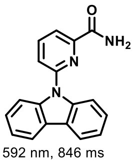
592 nm, 846 ms
Adv. Optical Mater. 2020, 2001685

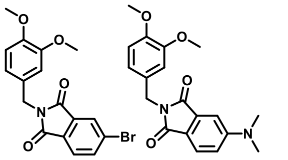
560 nm, 48 ms
Angew. Chem. Int. Ed. 2020, **59**, 10023-10026.

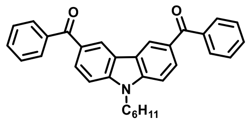
521 nm, 4000ms
Angew.Chem. Int. Ed. 2020, **59,** 13079-13085

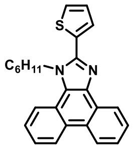
581 nm, 14.54 ms
Mater. Chem. Front., 2021,**5**, 817-824

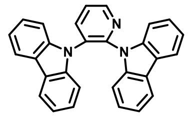
544 nm, 846 ms
Research, 2020, 9.

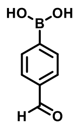
576 nm, 257 ms
J. Phys. Chem. A 2020, **124,** 2746-2754

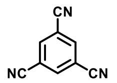
549 nm, 510 ms
J. Phys. Chem. C 2020, **124,** 10129-10134

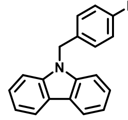
550 nm, 498 ms
J. Phys. Chem. Lett. 2020, **11,** 4962-4969

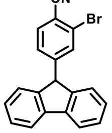
548 nm, 548 ms
Adv. Funct. Mater. 2021, 2101312

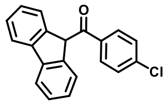
530 nm, 847 ms
Adv. Mater. 2017, 29, 1701244

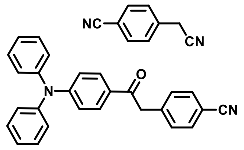
496 nm, 317 ms
J. Phys. Chem. Lett. 2019, **10,** 6019-6025

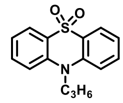
490 nm, 327 ms
Mater. Chem. Front., 2019, **3**, 1391-1397

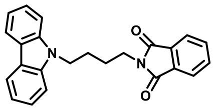
561 nm, 603 ms
Chem. Mater. 2019, **31,** 15, 5584-5591

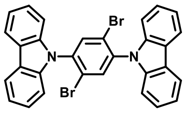
546 nm, 217 ms
J. Phys. Chem. Lett. 2019, **10,** 595-600

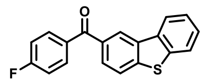
570 nm, 110 ms
Chem, **1**, 2016, 592-602.

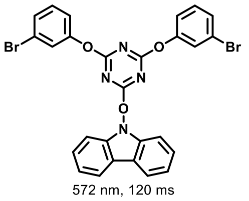
572 nm, 120 ms
Adv. Funct. Mater. 2018, **28,** 1705045

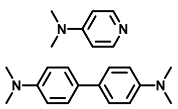
480 nm, 2010 ms
SmartMat. 2020, **1**, 1006.

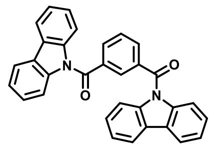
529 nm, 710 ms
Adv. Mater. 2019, **31,** 1807222

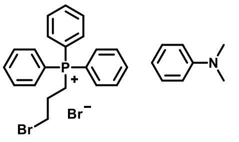
500 nm, 7h
Adv. Mater. 2020, 2001026

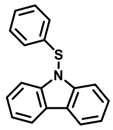
580 nm, 172 ms
J. Mater. Chem. C, 2020, **8**, 14740-14747

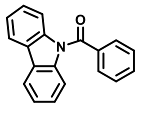
530 nm, 743 ms
Adv. Mater. 2017, **29,** 1606829

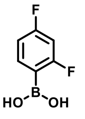
515 nm, 2500ms
Adv. Optical Mater. 2019, 1800820

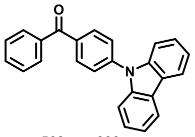
562 nm. 390ms
Angew. Chem. Int. Ed. 2016, **128,** 2221-2225

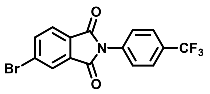
555 nm, 102 ms
Angew. Chem. Int. Ed. 2018, **130,** 6559-6563

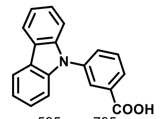
595 nm, 795 ms
Angew. Chem. Int. Ed. 2018, **130,** 8129-8133

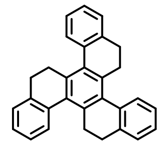
566 nm, 380 ms
Angew. Chem. Int. Ed. 2019, **131**, 7056-7060

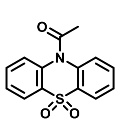
514 nm, 876 ms
Angew. Chem. Int. Ed. 2019, **58,** 6645-6649

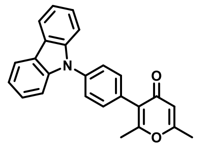
556 nm, 764 ms
J. Mater. Chem. C, 2020, **8**, 17410-17416.

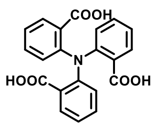
498 nm, 38 ms
Angew. Chem. Int. Ed. 2019, **58**, 17297-17302

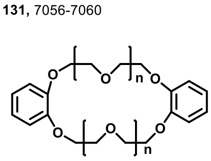
474 nm, 940 ms
Angew. Chem. Int. Ed. 2020, **59**, 9293-9298

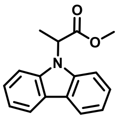

596 nm, 436 ms
Angew.Chem. Int. Ed. 2020, **59**, 4756-4762

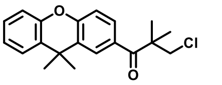

570 nm, 601 ms
Angew. Chem. Int. Ed. 2020, **59,** 9946-9951

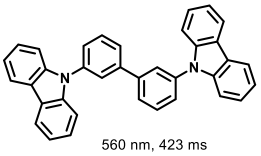

560 nm, 423 ms
Angew. Chem. Int. Ed. 2020, **59,** 10032-10036

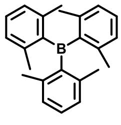

575 nm, 478 ms
Angew.Chem. Int. Ed. 2020, **59,** 17137-17144

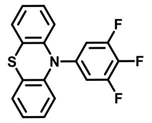

505 nm, 23.88ms
DOI: 10.1002/anie.202101994

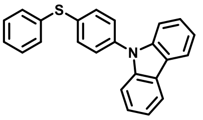

553 nm, 753 ms
Chem. Commun., 2018, **54,** 9226-9229

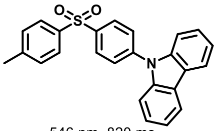

546 nm, 820 ms
Chem. Sci., 2019, **10**, 179-184

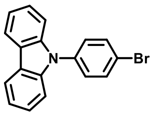

598 nm, 200 ms
Chem. Sci., 2019, **10,** 5031-5038

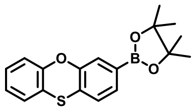

545 nm, 11.78 ms
Chem. Commun., 2019, **55,** 7215-7218

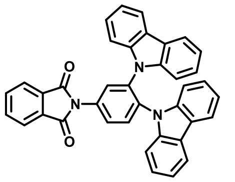

550 nm, 775 ms
Chem. Commun., 2020, **56**, 4296-4299

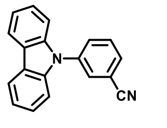

599 nm, 810 ms
Mater. Horiz., 2019, **6,** 1259-1264

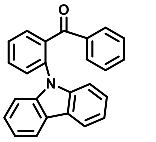

542 nm, 840 ms
Chem. Sci., 2019, **10,** 7352-7357

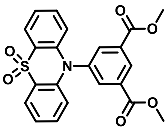

490 nm, 86 ms
Chem. Sci., 2020, **11,** 833-838

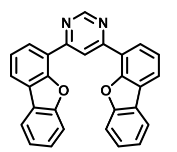

580 nm, 25.1 ms
J. Mater. Chem. C, 2020, **8,** 1871-1878

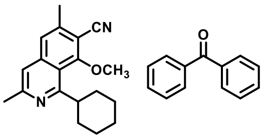

560 nm, 343 ms
Chem. Eur. J. 2020, **26,** 17376-17380

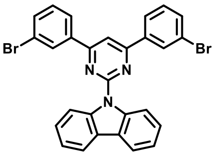

546 nm, 79 ms
Chem. Commun., 2020, **56**, 4051-4054

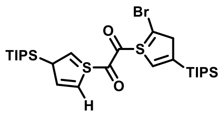

559 nm, 0.048ms
Chem. Commun., 2020, **56,** 6810-6813

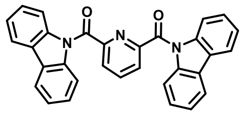

531 nm, 740 ms
Chem. Commun., 2020, **56,** 13559-13562

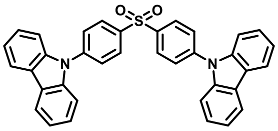

565 nm, 471 ms
Chem. Sci., 2021,**12,** 188-195

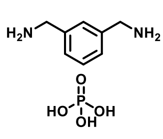

514 nm, 376 ms
J. Mater. Chem. C, 2020, **8**, 7330-7335

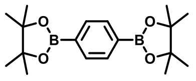

500 nm, 1820 ms
J. Am. Chem. Soc. 2017, **139, 2728-2733**

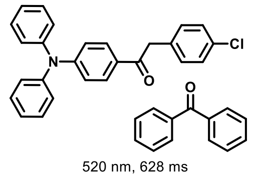

520 nm, 628 ms
J. Mater. Chem. C, 2021, **9**, 3391-3395

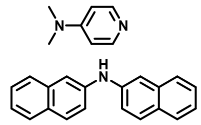

530 nm, 766 ms
Matter. 2020, **3,** 449-463

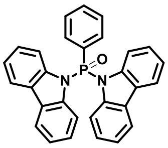

582 nm, 660 ms
Adv. Mater. 2018, **30,** 1803856

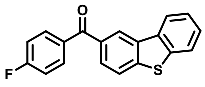

600 nm, 107 ms
Nat. Commun. 2017, **8,** 416

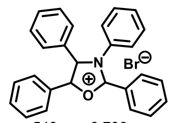

549 nm, 0.706 ms
Nat. Commun. 2018, **9,** 2963.

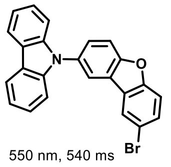

550 nm, 540 ms
Nat. Commun. 2019, **10,** 1595.

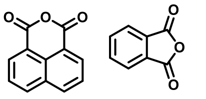

540 nm, 603 ms
Nat. Commun. 2019, **10**, 5161

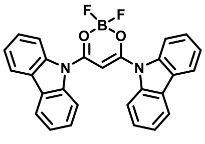

525 nm, 230 ms
Nat. Commun. 2020, **11,** 842.

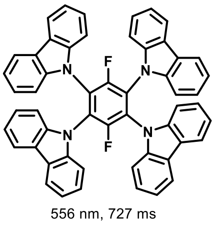

556 nm, 727 ms
Nat. Commun. 2020, **11,** 2617.

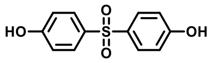

488 nm, 828 ms
Nat. Commun. 2021, **12,** 2297.

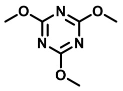

505 nm, 504 ms
Nat. Photonics 2019, **13,** 406-411.

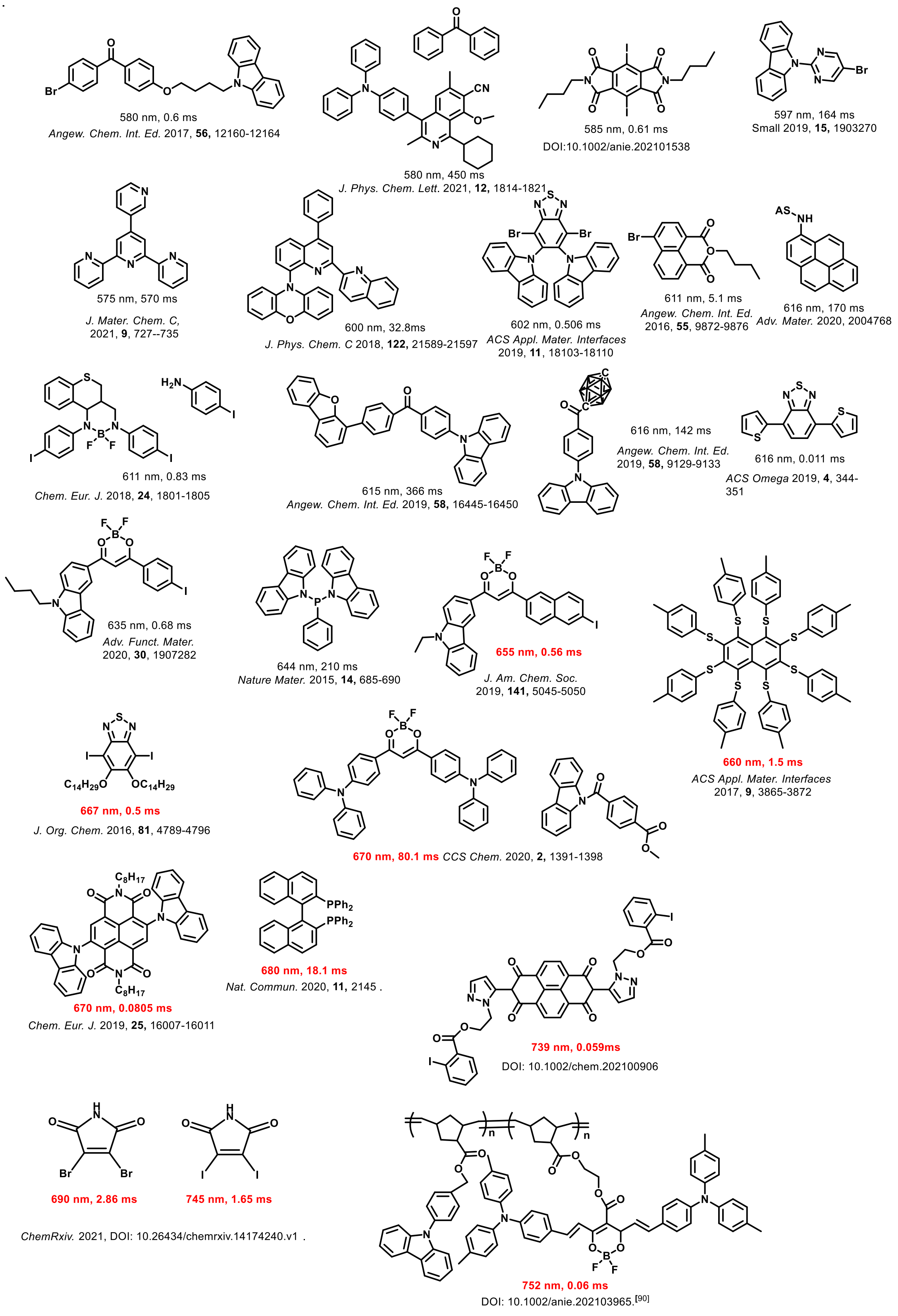

Supplementary Fig. 1. Structural formula. Organic RTP materials that have been reported.

2. Synthesis of guest compounds and preparation of doped materials

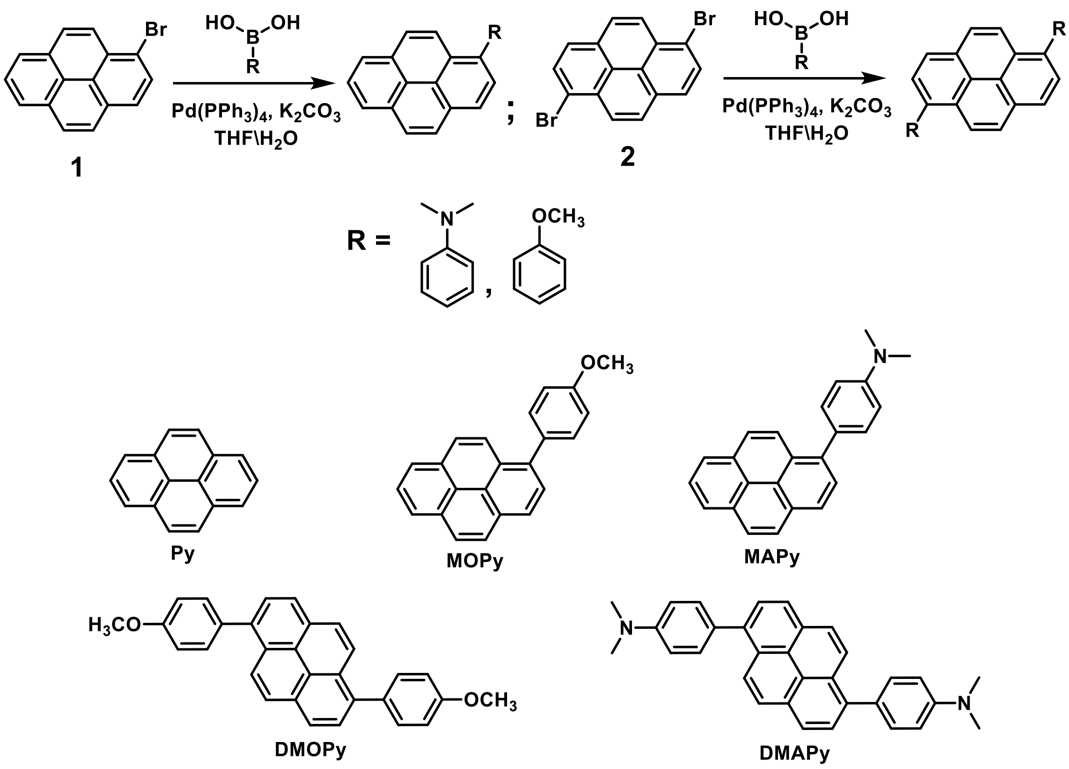

Supplementary Fig. 2. Synthetic route. Synthetic routes of five guest compounds.

3. Experimental data

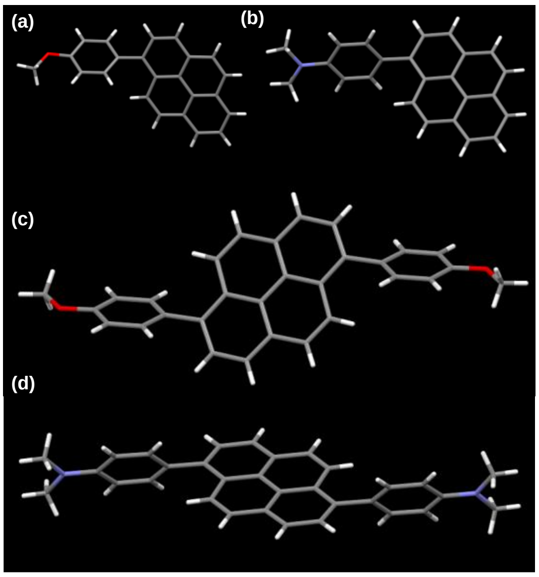

Supplementary Fig. 3. Single crystal. Single crystal structures of **MOPy** (a), **MAPy** (b), **DMOPy** (c), **DMAPy** (d). (CCDC: **MOPy**: 2091331, **MAPy**: 2091334, **DMOPy**: 2091335, **DMAPy**: 2091336)

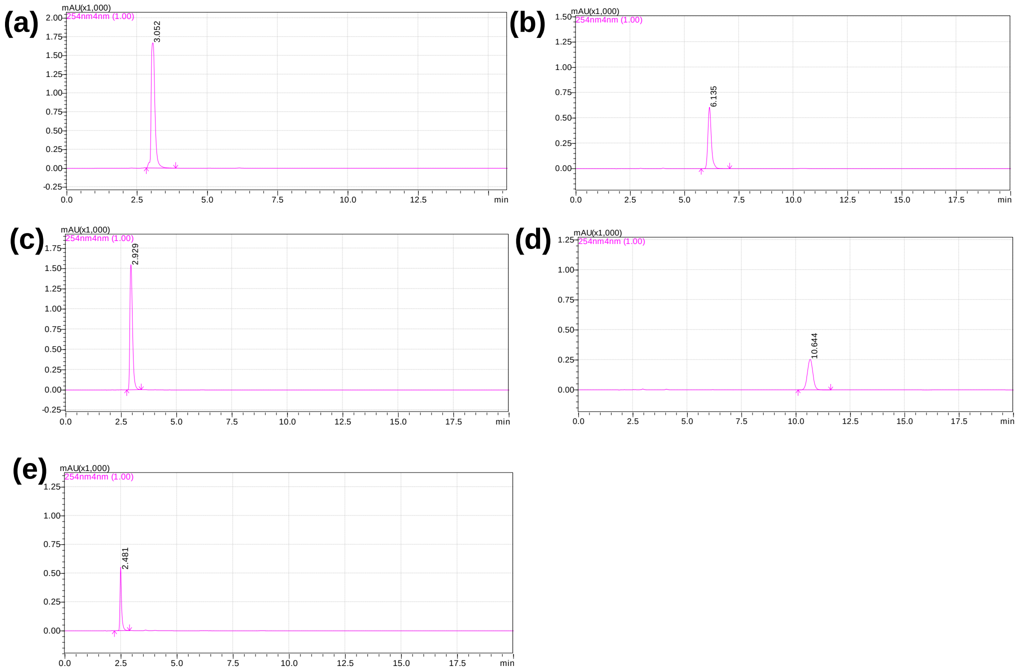

Supplementary Fig. 4. High performance liquid chromatography. HPLC spectra of **Py** (a), **MOPy** (b), **MAPy** (c), **DMOPy** (d), **DMAPy** (e) (CH_3OH/H_2O= 80%: 20%)

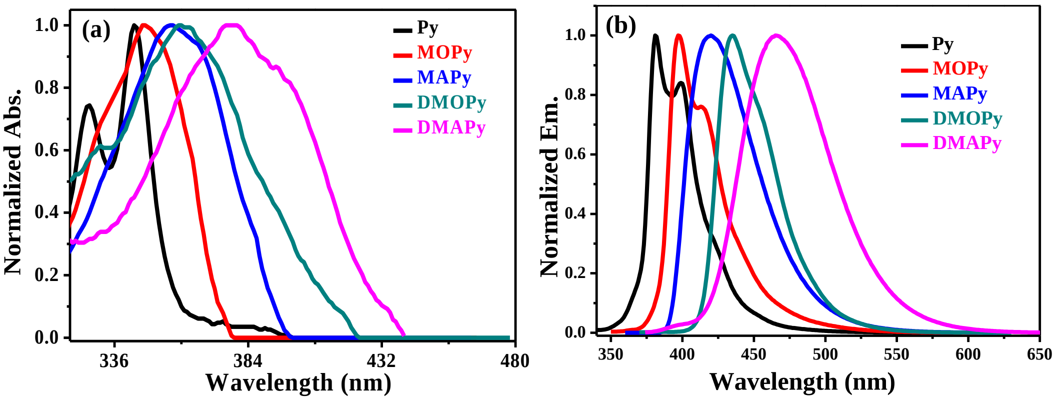

Supplementary Fig. 5. **Absorption and emission spectra**. (a) Normalized absorption spectra of the guests in THF solvent. (b) Normalized emission spectra of the guests in THF solvent. Concentration: 1.0×10^{-5} mol/L.

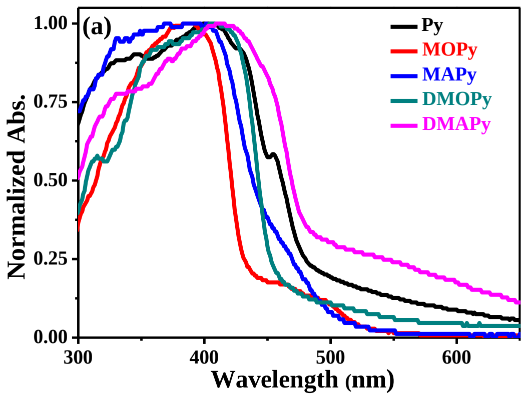

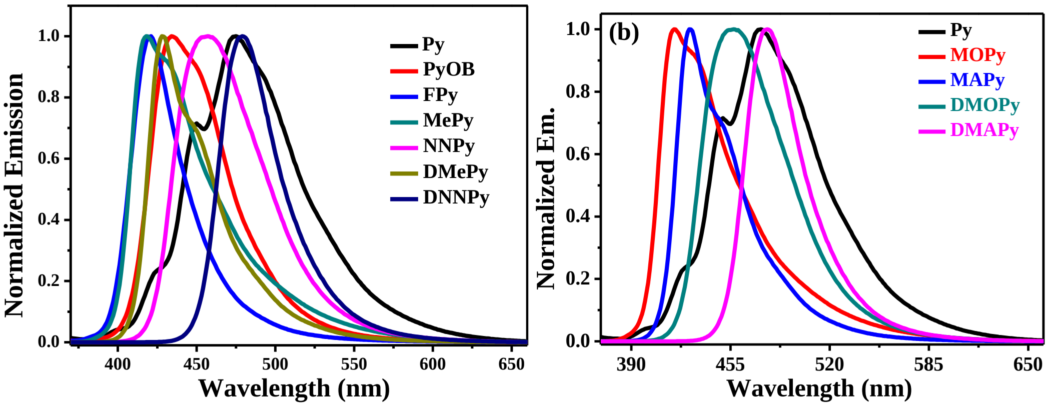

Supplementary Fig. 6. **Absorption and emission spectra**. (a) Normalized absorption spectra of the guests in solid state. (b) Normalized emission spectra of the guests in solid state.

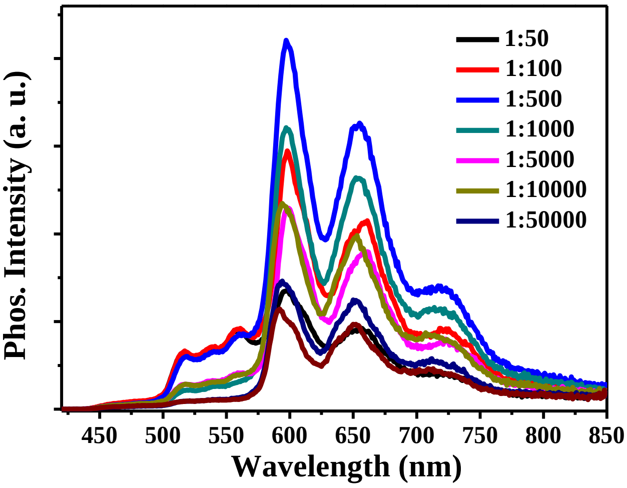

Supplementary Fig. 7. **Emission spectra.** Delayed emission spectra of **Py/BPO** guest-host materials with different amounts of **Py**. (Delayed time: 1 ms, Ex.: 380 nm).

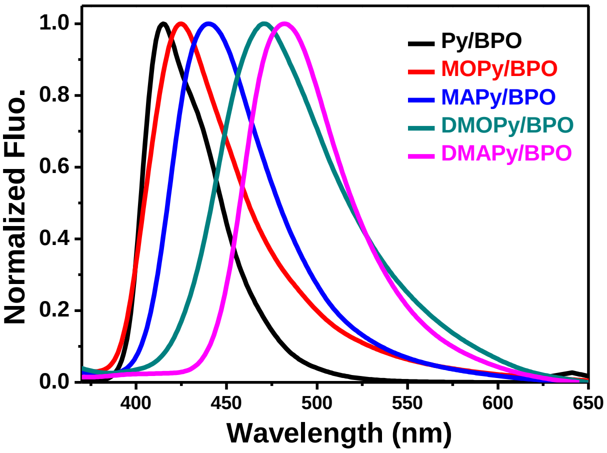

Supplementary Fig. 8. **Emission spectra.** Fluorescence spectra of guest-host materials. (Ex.: 360 nm)

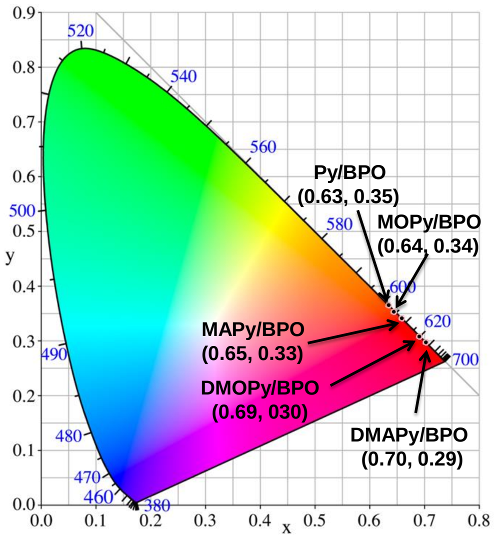

Supplementary Fig. 9. **CIE coordinate**. CIE coordinates of delayed emission of guest-host materials.

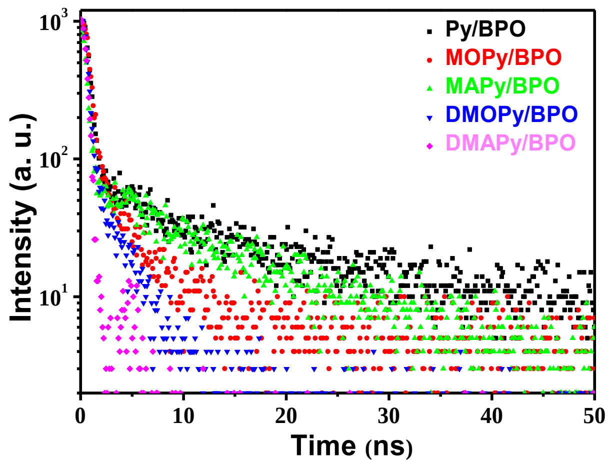

Supplementary Fig. 10. **Decay curves.** Fluorescence decay curves of guest-host materials. (Ex.: 360 nm)

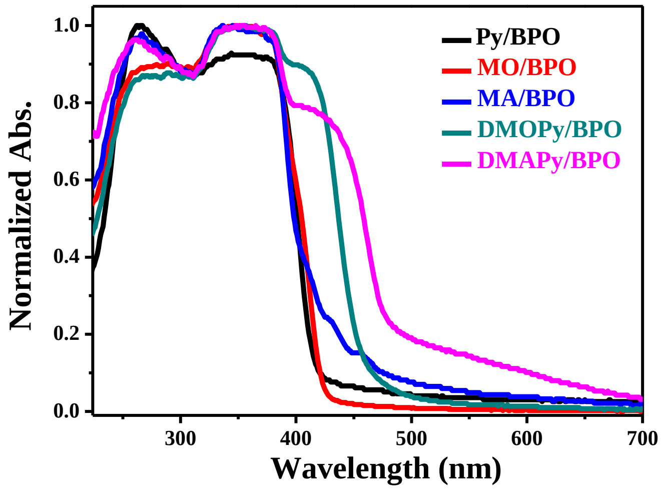

Supplementary Fig. 11. **Absorption spectra**. Absorption spectra of guest-host materials.

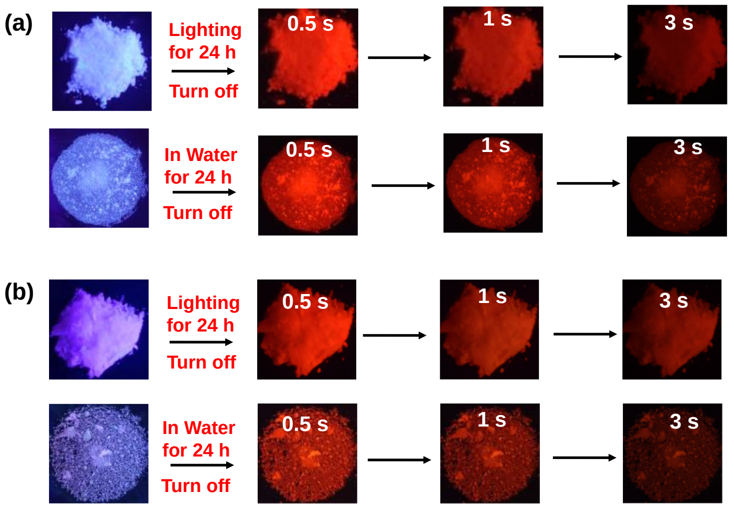

Supplementary Fig. 12. Luminous photograph. (a) Phosphorescence photographs of **Py/BPO** in different state. (b) Phosphorescence photographs of **MAPy/BPO** in different state.

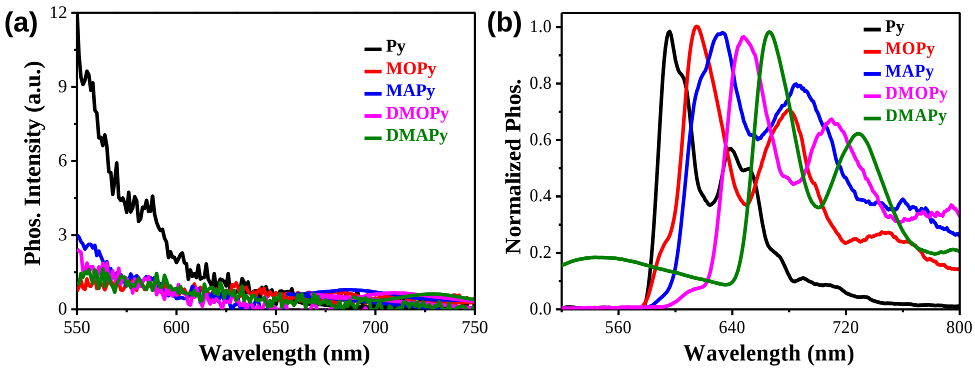

Supplementary Fig. 13. Emission spectra. (a) Phosphorescence spectra of the solid guests in 77 K (Ex: 380 nm; Delayed time: 1 ms) (b) Phosphorescence spectra of the solution guests in 77 K (Delayed time: 1 ms; Ex: 380 nm; Concentration: 1×10^{-5} mol/L; Solvent: 2-methyltetrahydrofuran).

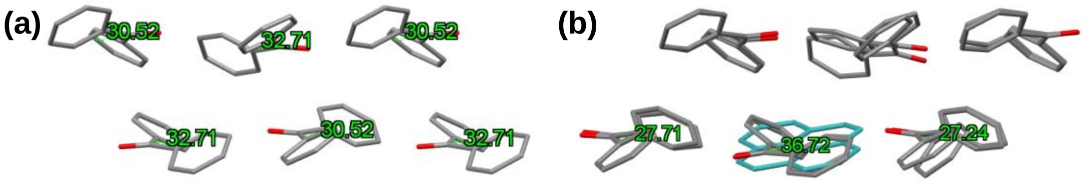

Supplementary Fig. 14. **Molecular configuration**. (a) The molecular configuration of **BPO** in single crystal. (b) The molecular configuration of **BPO** in simulated **Py/BPO** system.

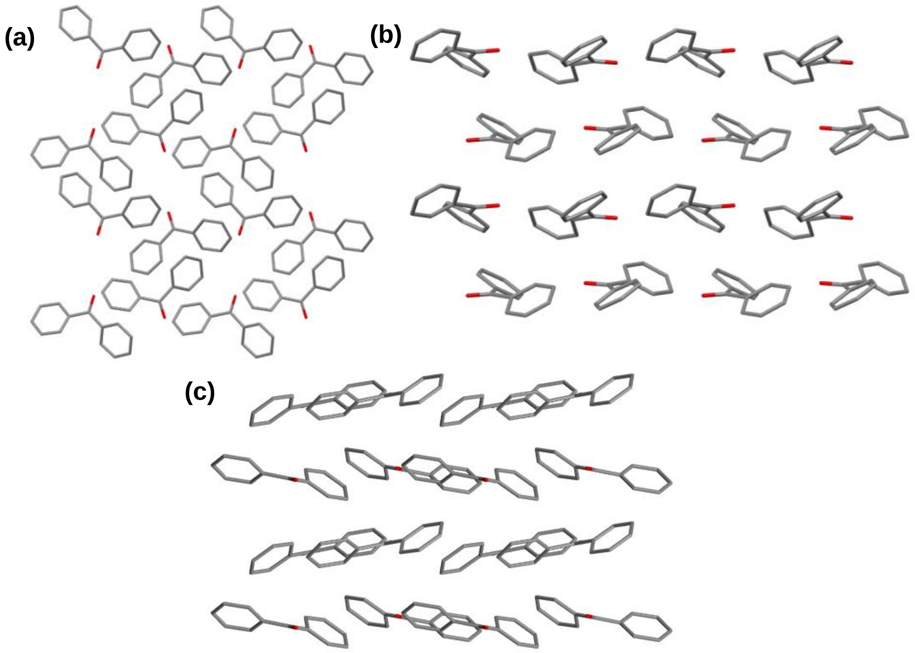

Supplementary Fig. 15. **Molecular arrangement**. Molecular packing along the a-axis (a), b-axis(b), c-axis(c) of **BPO** single crystal.

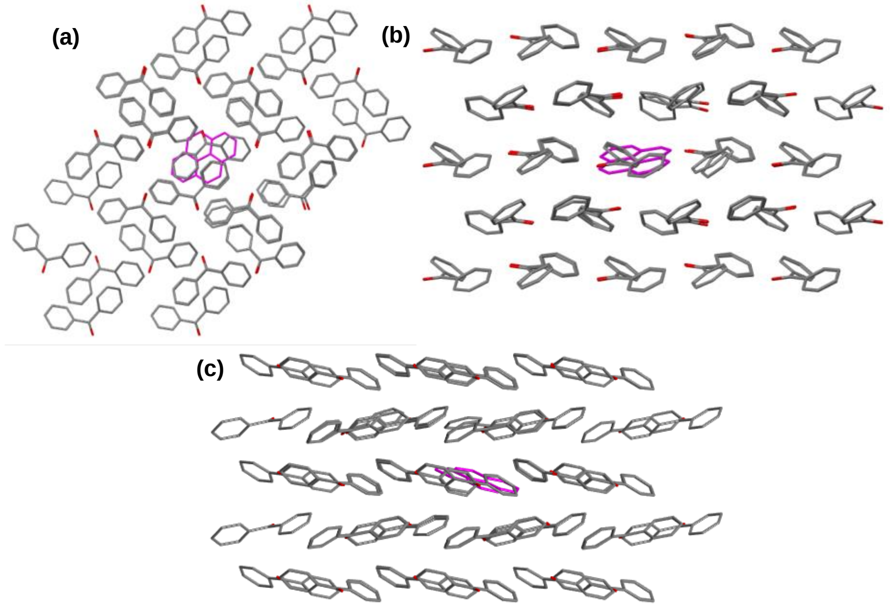

Supplementary Fig. 16. **Molecular arrangement.** Molecular packing along the a-axis (a), b-axis(b), c-axis(c) of simulated **Py/BPO** guest-host system.

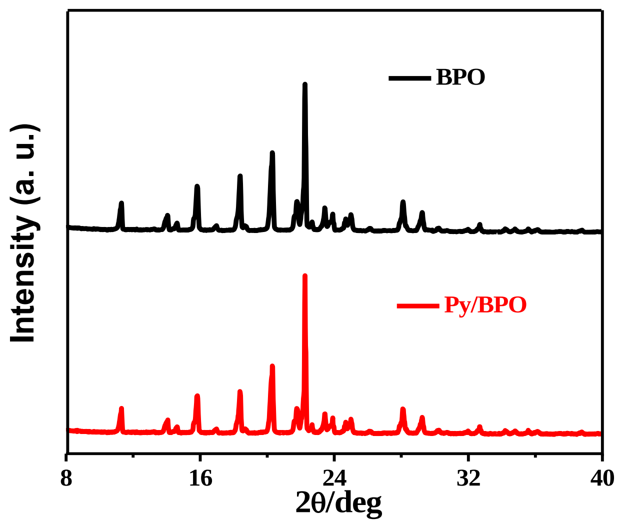

Supplementary Fig. 17. **XRD spectra**. The XRD curves of **BPO** and **Py/BPO** doped material.

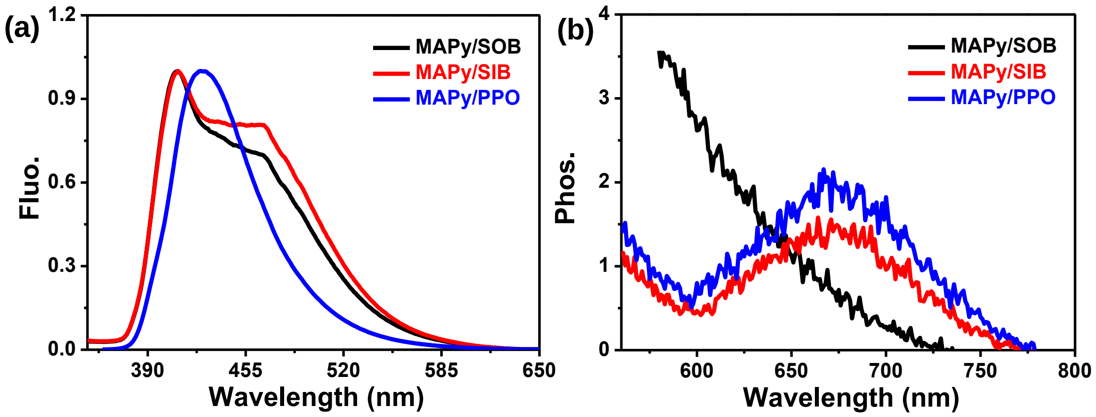

Supplementary Fig. 18. **Emission spectra**. Fluorescence (a) and phosphorescence (b) spectra of the reference guest-host materials. Ex. of Phos.: 380 nm; Delayed time: 1 ms.

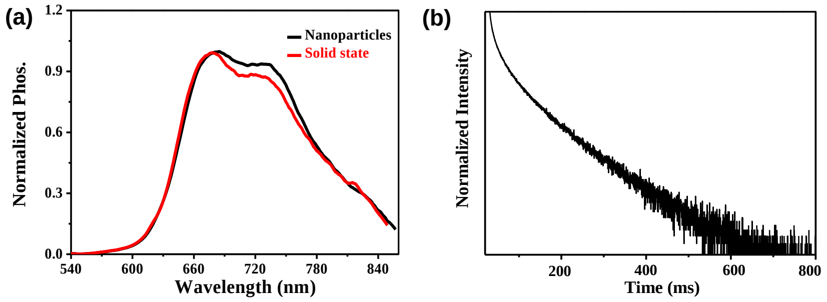

Supplementary Fig. 19. Emission and decay spectra. (a) Phosphorescence spectra of the **DMAPy/BPO** in nanoparticles state and solid state. (b) Phosphorescence decay curves of **DMAPy/BPO** nanoparticles.

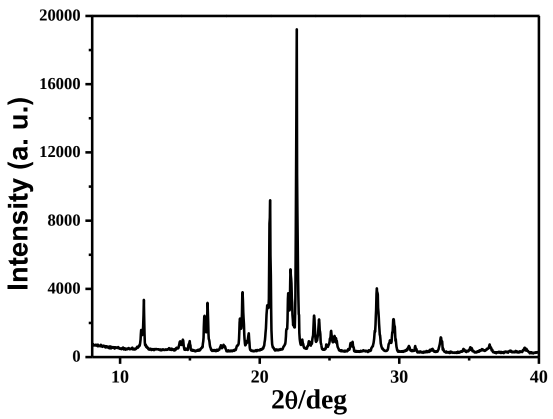

Supplementary Fig. 20. XRD spectra. The XRD curve of **DMAPy/BPO** nanoparticles.

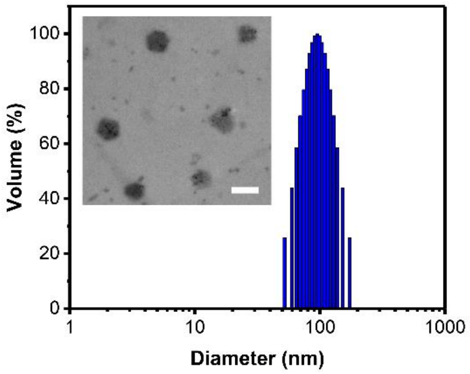

Supplementary Fig. 21. Diameter distribution. Diameter distribution of **DOB/BPO** nanoparticles. Inset: TEM image, scale bar = 100 nm.

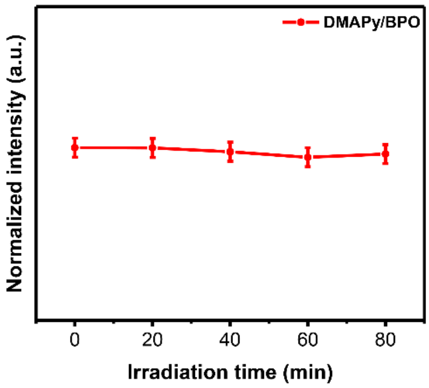

Supplementary Fig. 22. Anti-photobleaching capability. The phosphorescence intensities for **DMAPy/BPO** nanoparticles with varying UV exposure time. Error bars, mean ±standard deviation (n = 3).

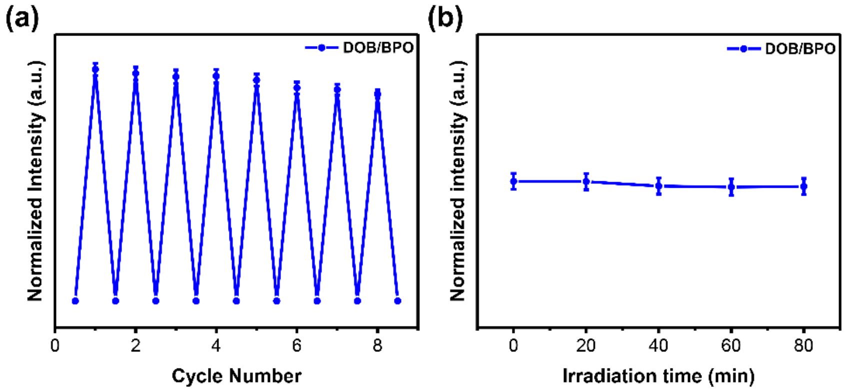

Supplementary Fig. 23. Anti-fatigue and anti-photobleaching capability. (a) The phosphorescence intensities of **DOB /BPO** nanoparticles as a function of the cycle number of UV light irradiation (n = 3). (b) The phosphorescence intensities for **DOB /BPO** nanoparticles with varying UV exposure time. Error bars, mean ±standard deviation (n = 3).

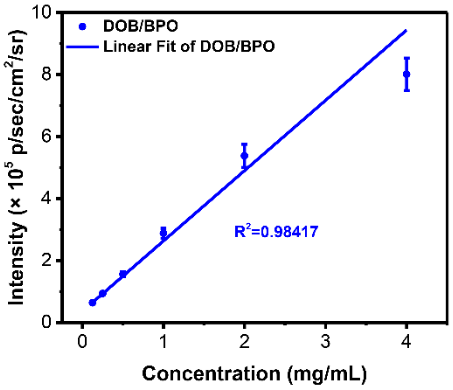

Supplementary Fig. 24. Phosphorescence intensity linearity. The phosphorescence intensities as a function of the concentration of **DMAPy/BPO** nanoparticles (n = 3).

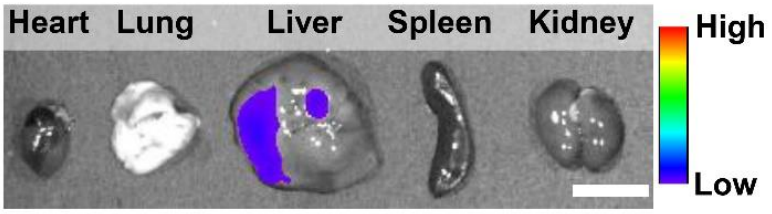

Supplementary Fig. 25. Tissue distribution. The phosphorescence signals of different organs (1-heart, 2-liver, 3-spleen, 4-lung and 5-kidney) from tumor-bearing mice at 6 h post intravenous injection of DMAPy/BPO nanoparticles. scale bar=1 cm.

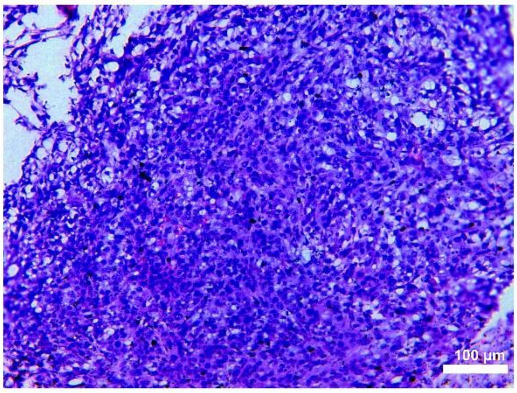

Supplementary Fig. 26. Tumor H&E-stained image. H&E-stained image indicated that the light-up tissue was tumor.

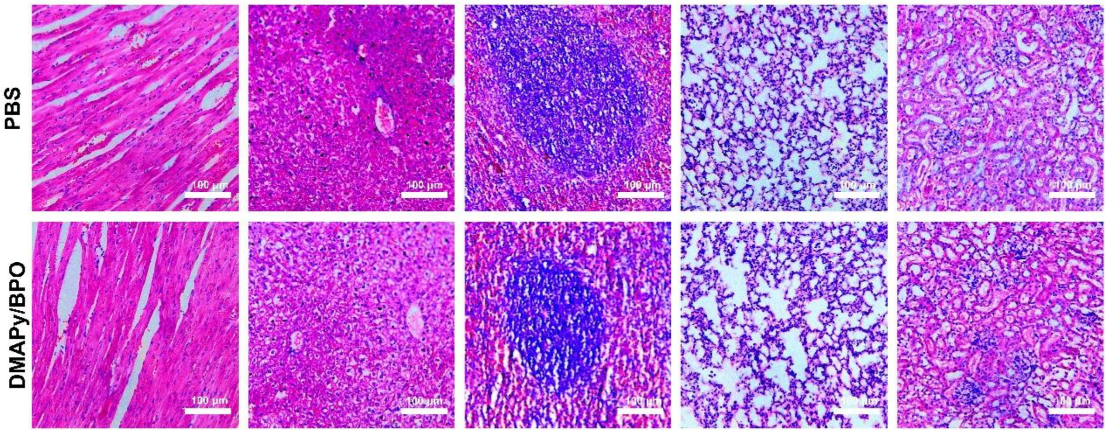

Supplementary Fig. 27. Major organs H&E-stained images. H&E-stained images of major organs of the tumor-bearing mice after intravenous injection of saline and **DMAPy/BPO** nanoparticles, respectively.

4. NMR spectra

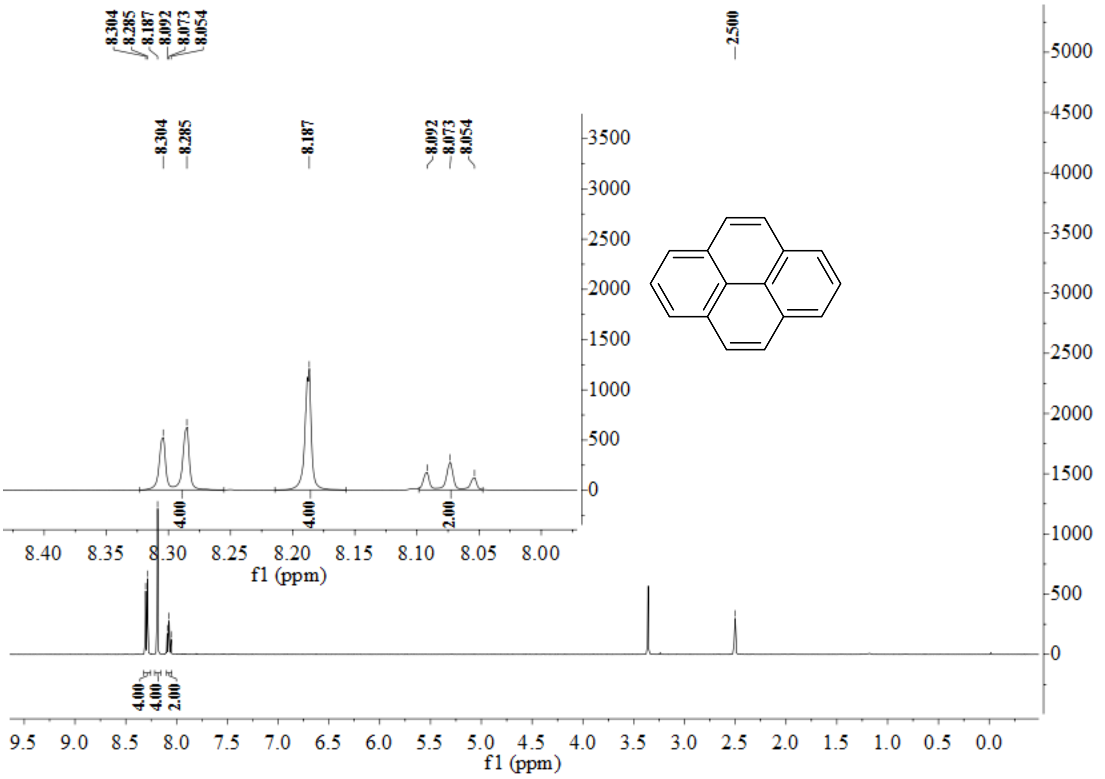

Supplementary Fig. 28. **NMR spectrum.** ^{1}H NMR of compound **Py** (DMSO-d_6, 400 MHz).

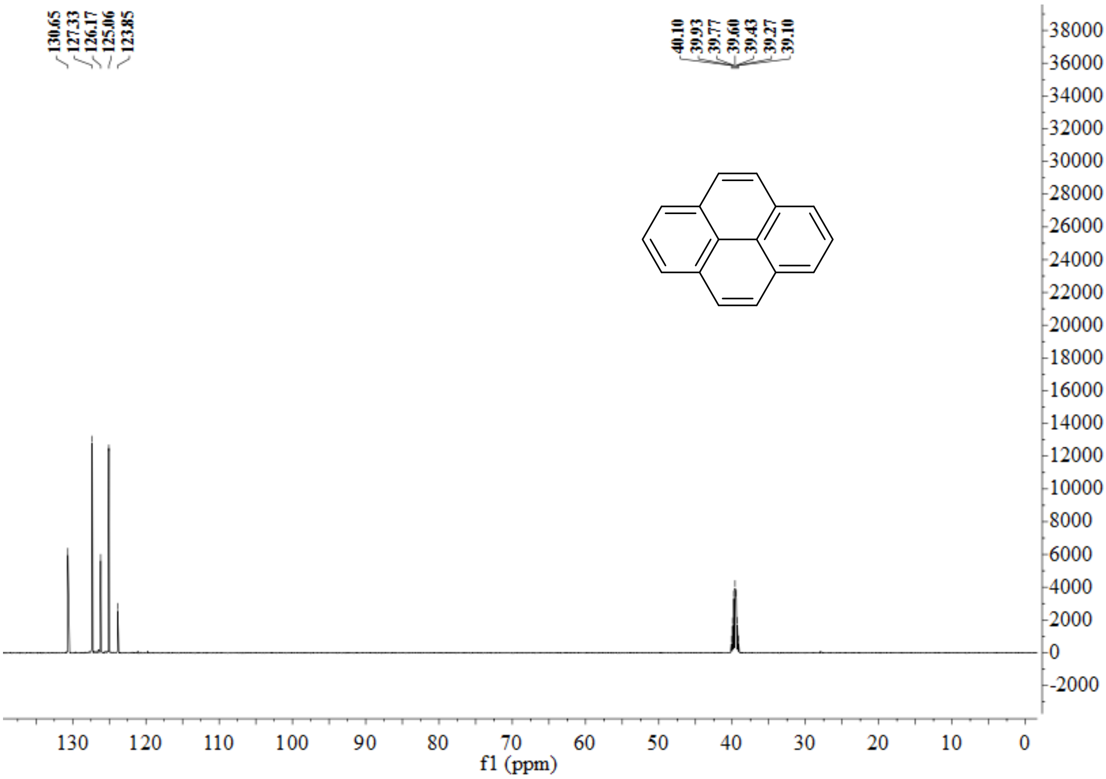

Supplementary Fig. 29. **NMR spectrum.** ^{13}C NMR of compound **Py** (DMSO-d_6, 125 MHz).

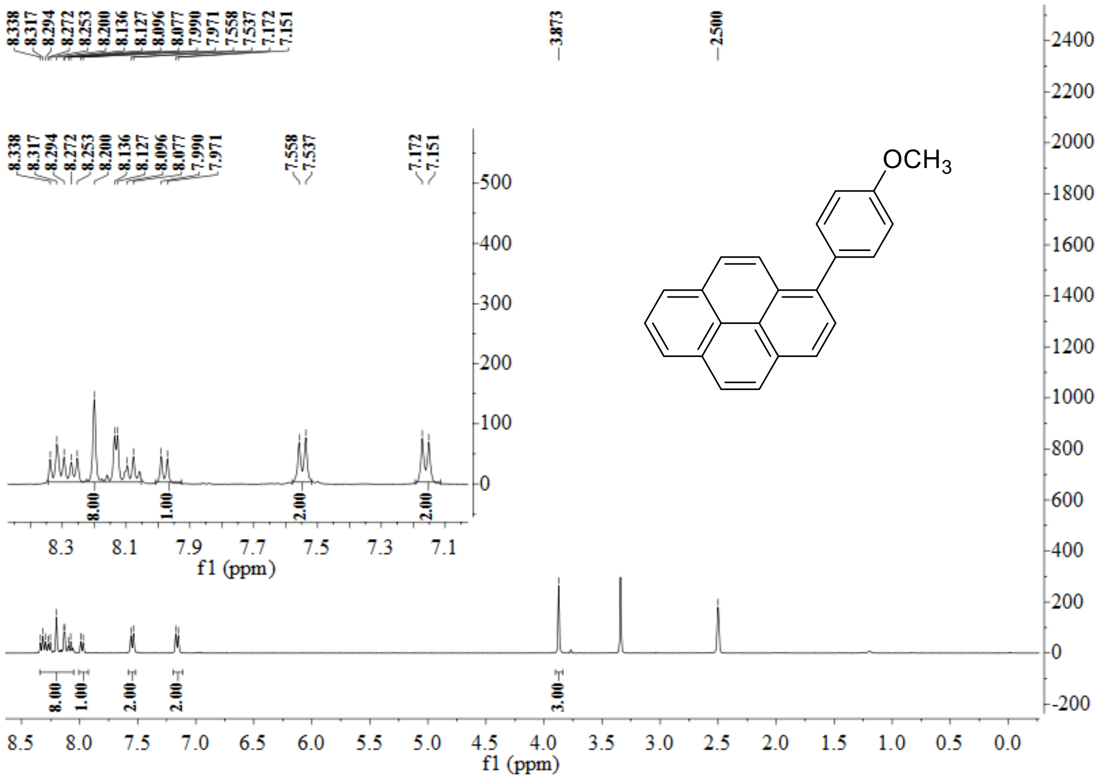

Supplementary Fig. 30. **NMR spectrum.** ^{1}H NMR of compound **MOPy** (DMSO-d_6, 400 MHz).

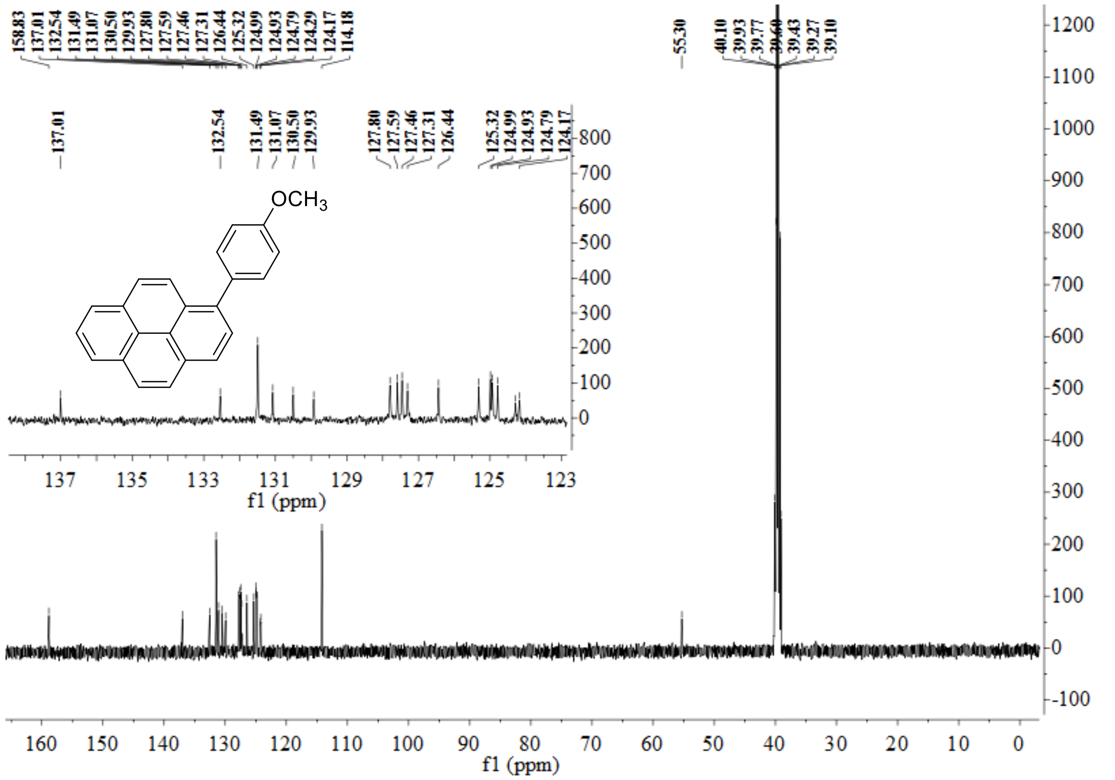

Supplementary Fig. 31. **NMR spectrum.** ^{13}C NMR of compound **MOPy** (DMSO-d_6, 125 MHz).

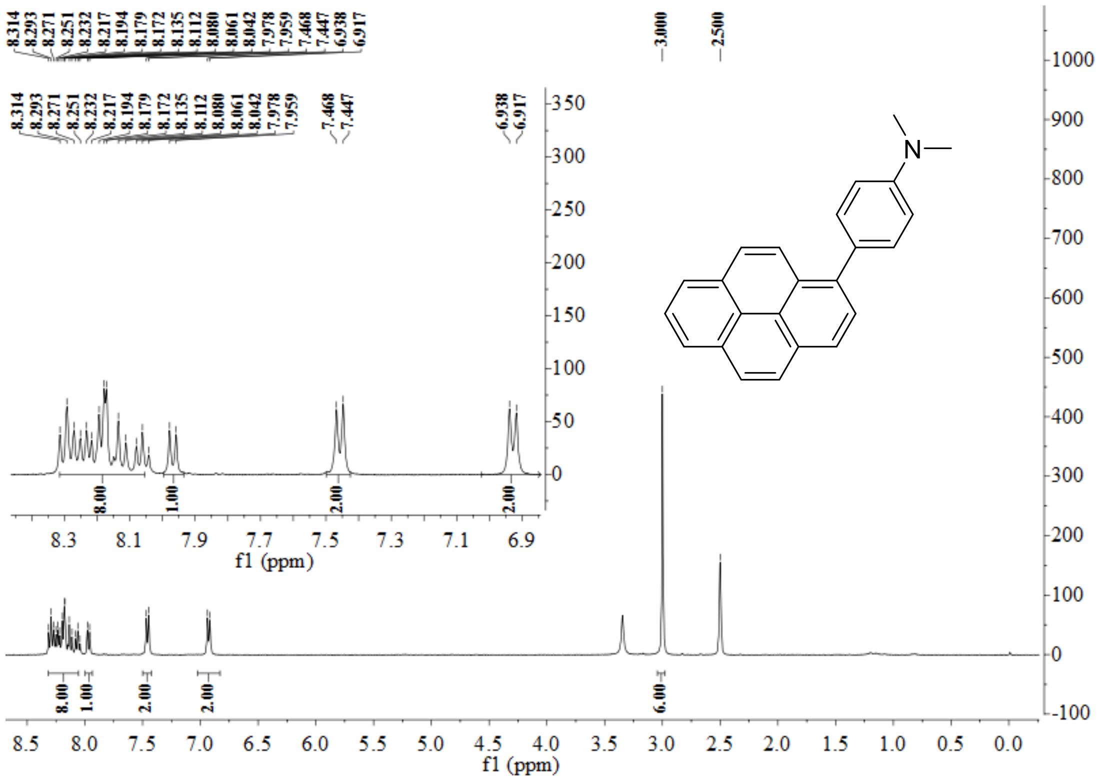

Supplementary Fig. 32. **NMR spectrum.** ^{1}H NMR of compound **MAPy** (DMSO-d_6, 400 MHz).

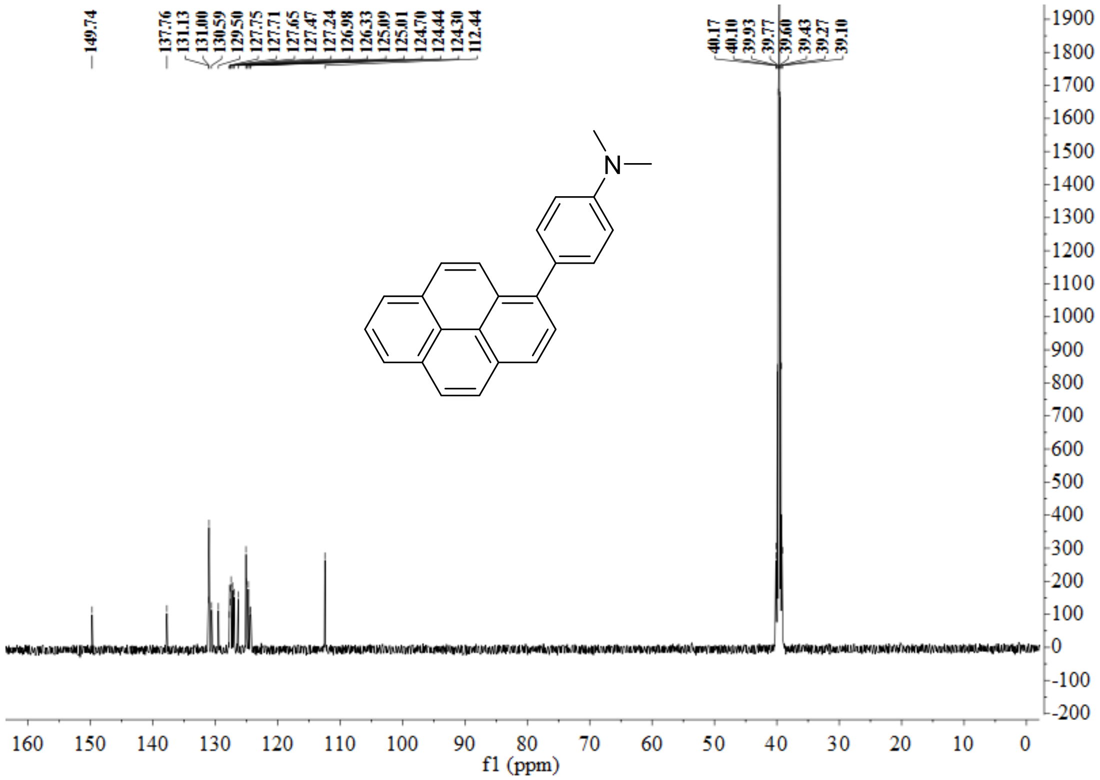

Supplementary Fig. 33. **NMR spectrum.** ^{13}C NMR of compound **MAPy** (DMSO-d_6, 125 MHz).

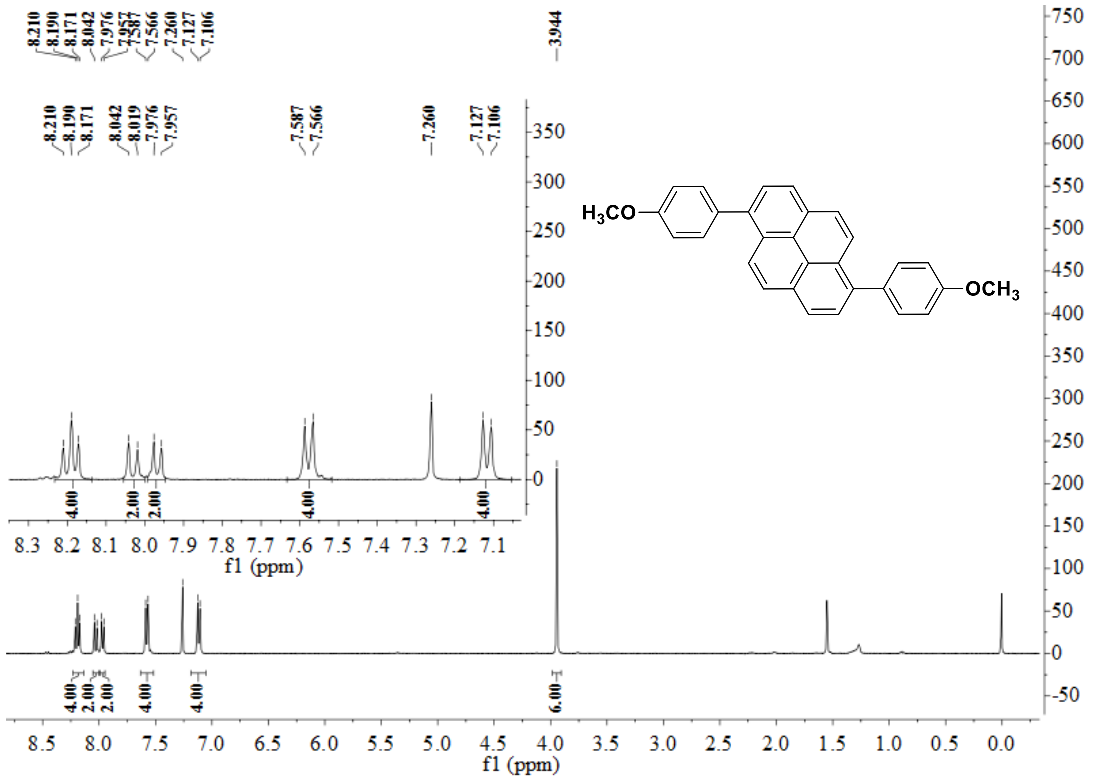

Supplementary Fig. 34. **NMR spectrum.** ^{1}H NMR of compound **DMOPy** ($CDCl_3$, 400 MHz).

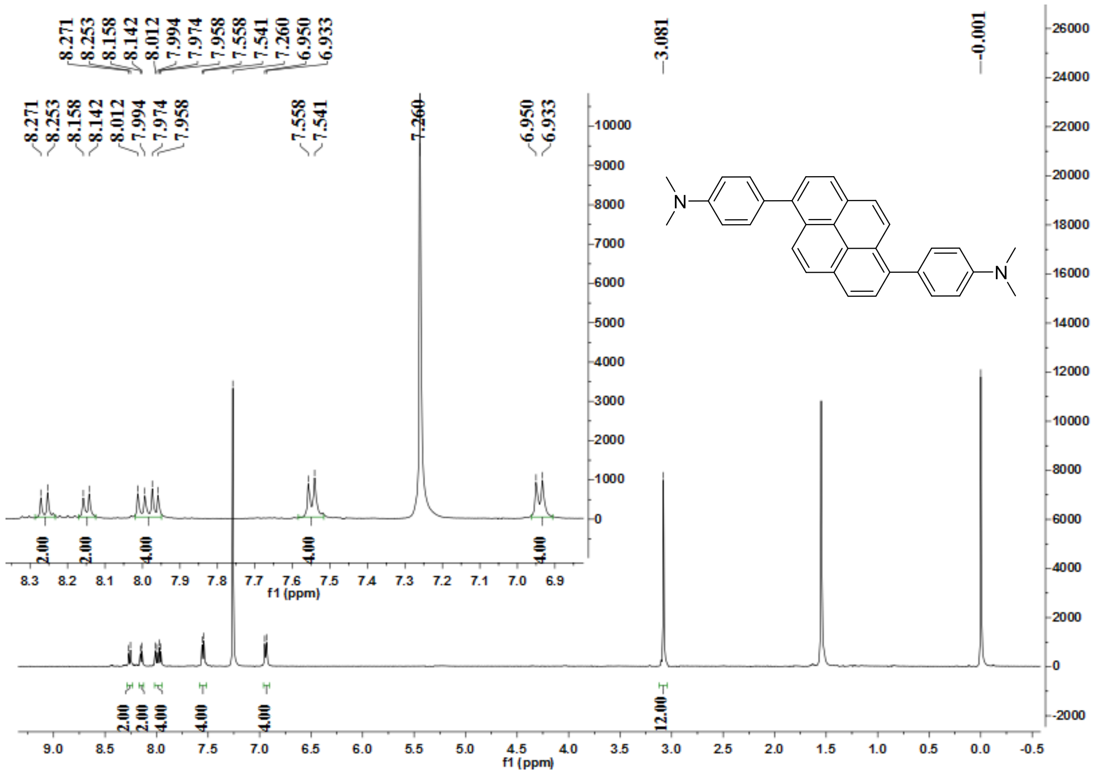

Supplementary Fig. 35. **NMR spectrum.** ^{1}H NMR of compound **DMAPy** ($CDCl_3$, 500 MHz).

Supplementary Methods

1. Materials, Characterization and Molecular dynamics (MD) simulations.

^{1}H and ^{13}C NMR spectra were carried out by a Bruker ARX500 spectrometer with $CDCl_3$ as the solvent. UV-vis absorption spectra were measured by a Persee TU-1901 spectroscopy. Fluorescence spectra were measured by a Hitachi F-7000 spectrophotometer. Phosphorescence spectra were measured by a FLS920 lifetime and steady state spectrometer. X-Ray crystal structure analyses were conducted on a Bruker-AXS SMART APEX2 CCD diffractometer. Solid-state emission quantum yields (Φ) were collected on a FluoroMax-4 (Horiba Jobin Yvon) fluorimeter equipped with integrated sphere. The emission decay data were analyzed using DAS-6 Fluorescence Decay Analysis software with 2 exponential analysis the program. The quality of the exponential fits was evaluated by the $\chi 2$ of <2.0.

The atom types and parameters of **Py** and **BPO** were built from the general AMBER force field. To obtain the partial charge of each atom of two molecules, the electrostatic potential of two molecules were calculated at the B3LYP/6-31G* level based on the optimized geometric structures using Gaussian 16 package1. The partial charges of the atoms reproducing the electrostatic potential of two molecules were obtained by using restrained electrostatic potential (RESP)2, 3 fit methods. In order to simulate **Py/BPO** system, we firstly built a 4.6×3.1×4.4 nm TPA unit cell with 192 TPA molecules, and then a **Py** molecule was placed in the position vacated by the one host molecules to obtain the **Py/BPO** model as an initial model. For MD simulations, firstly, the energy minimization was performed by using steepest descent algorithm and the conjugate gradient, then we performed the 500 ps MD simulations under the NVT (P = 1 bar, T = 10 K) ensemble. The temperature was controlled by the velocity rescaling thermostat4. Then we performed the 10 ns MD simulations under the NPT (P = 1 bar, T = 10 K) ensemble coupled by Parrinello-Rahman barostat. The Newton's classical equations of motion were integrated at a time step of 2 fs using the classical leapfrog algorithm. Trajectory analysis was done with the help of utility tools included in the GROMACS (version 5.1.5)6 and VMD7 packages.

2. Synthesis of pyrene.

The Pyrene compound is directly purchased commercially and purified by column chromatography twice. Pyrene (**Py**): ^{1}H NMR (400 MHz, DMSO-d_6) δ: 8.29 (d, J = 7.6 Hz, 4H), 8.19 (s, 4H), 8.07 (d, J = 7.6 Hz, 2H), ppm. ^{13}C NMR (125 MHz, DMSO-d_6) δ: 130.7, 127.3, 126.2, 125.1, 123.9 ppm. HRMS (ESI) m/z: $[M+H]^+$ calculated for $C_{16}H_{11}$, 203.0863; found, 203.0855.

3. Characterization data of pyrene derivatives.

1-(4-Methoxyphenyl)pyrene (MOPy): White solid (2.22 g, 72%). ^{1}H NMR (400 MHz, DMSO-d_6) δ: 8.34-8.08 (m, 8H), 7.98 (d, J = 7.6 Hz, 1H), 7.55 (d, J = 8.4 Hz, 2H), 7.16 (d, J = 8.4Hz, 2H), 3.87 (s, 3H) ppm. ^{13}C NMR (125 MHz, DMSO-d_6) δ: 158.8, 137.0, 132.5, 131.5, 131.1, 130.5, 129.9, 127.8, 127.6, 127.5, 127.3, 126.4, 125.3, 125.0, 124.9, 124.8, 124.3, 124.2, 114.2, 55.3 ppm. HRMS (ESI) m/z: $[M+H]^+$ calculated for $C_{23}H_{17}O$, 309.1274; found, 309.1278.

***N,N*-Dimethyl-4-(pyren-1-yl)aniline (MAPy):** Green solid (2.09 g, 65%). ^{1}H NMR (400 MHz, DMSO-d_6) δ: 8.31-8.04 (m, 8H), 7.97 (d, J = 7.6 Hz, 1H), 7.46 (d, J = 8.4 Hz, 2H), 6.93 (d, J = 8.4 Hz, 2H), 3.00 (s, 6H) ppm. ^{13}C NMR (125 MHz, $CDCl_3$) δ: 149.7, 137.8, 131.1, 131.0, 130.6, 129.5, 127.8, 127.7, 127.65, 127.5, 127.2, 127.0, 126.3, 125.1, 125.0, 124.7, 124.4, 124.3, 112.4 ppm. HRMS (ESI) m/z: $[M+H]^+$ calculated for $C_{24}H_{19}N$, 322.1590; found, 322.1583.

1,6-Bis(4-methoxyphenyl)pyrene (DMOPy): Green solid (2.28 g, 55%). ^{1}H NMR (400 MHz, $CDCl_3$) δ: 8.19 (t, J = 8.0 Hz, 4H), 8.03 (d, J = 9.2 Hz, 2H), 7.97 (d, J = 8.0 Hz, 2H), 7.58 (d, J = 8.4 Hz, 4H), 7.12 (d, J = 8.4 Hz, 4H), 3.94 (s, 6H) ppm. HRMS (ESI) m/z: $[M+H]^+$ calculated for $C_{30}H_{23}O_2$, 415.1693; found, 415.1691.

4,4'-(Pyrene-1,6-diyl)bis(*N,N*-dimethylaniline) (DMAPy): Green solid (2.33 g, 53%). ^{1}H NMR (500 MHz, $CDCl_3$) δ: 8.26 (d, J = 9.0 Hz, 2H), 8.15 (d, J = 8.0 Hz, 2H), 7.99 (q, J = 9.0 Hz, 4H), 7.55 (d, J = 8.5 Hz, 4H), 6.94 (d, J = 8.5 Hz, 4H), 3.08 (s, 12H) ppm. HRMS (ESI) m/z: $[M+H]^+$ calculated for $C_{32}H_{29}N_2$, 441.2325; found, 441.2334.

4. Cell Culture.

4T1 breast cancer cells were obtained from American Type Culture Collection (ATCC). The cells were cultured in DMEM (Dulbecco's Modified Eagle Medium) (GIBCO) supplemented with 10% FBS and 1% penicillin streptomycin at 37 °C in a humidified environment containing 5% CO2, which were regularly checked for mycoplasma contamination.

5. Cytotoxicity Study.

In vitro cytotoxicities of the nanocrystals against cancer cells were assessed by 3-(4,5-dimethylthiazol-2-yl)-2,5-diphenyl tetrazolium bromide (MTT) assay. Briefly, 4T1 breast cancer cells seeded in 96-well plates (Costar, IL, USA) were exposed to each kind of nanocrystals at the RTP dye concentrations of 25, 50, 100, 200, 400 μg mL^{-1}, respectively, at 37 °C. After 8 h incubation, the wells were washed twice with 1×PBS, and 100 μL of freshly prepared MTT (0.5 mg mL^{-1}) solution in culture medium was added into each well. The MTT medium solution was carefully removed after 3 h incubation in the incubator. DMSO (100 μL) was then added into each well and the plate was gently shaken for 10 min at room temperature to dissolve all the precipitates formed. The absorbance of MTT at 490 nm was monitored by the microplate reader (Genios Tecan). Cell viability was expressed by the ratio of absorbance of the cells incubated with nanoparticles suspension to that of the cells incubated with culture medium only.

6. Animals and tumor models.

Six-weeks-old BALB/c nude mice were purchased from the Laboratory Animal Center of the Academy of Military Medical Sciences (Beijing, China).

To establish the armpit tumor-bearing mouse model, 5×10^5 4T1 cancer cells in 100 μL of cell culture medium were injected into the armpit of healthy mice. After about 7 days, the armpit tumor was formed.

7. Statement of ethical approval

All animal studies were performed according to the guidelines set by the Tianjin Committee of Use and Care of Laboratory Animals, and the overall project protocols were approved by the Animal Ethics Committee of Nankai University. The accreditation number of the laboratory is SYXK(Jin) 2019-0003 promulgated by

8. Subcutaneous phosphorescent imaging with nanoparticles. The healthy nude mice were

anesthetized using 2% isoflurane in oxygen and placed into the box of IVIS® instrument. Then, 100 μL of the **DMAPy/BPO** and **DOB/BPO** nanoparticles were subcutaneously injected into the mice, respectively, followed by irradiating by 365 nm hand-held UV lamp (12 W) for 1 min and imaging with IVIS® instrument. During the imaging process, the mice were warmed with a heating pad under continued isoflurane anesthesia. The phosphorescent images were acquired in bioluminescence mode with open filter setting (exposure time: 17 s) and in fluorescence mode with Dsred filter setting (excitation: 430 nm, exposure time: auto).

9. Phosphorescent imaging of sentinel lymph node. The healthy nude mice were anesthetized using 2% isoflurane in oxygen and placed into the box of IVIS® instrument. Then, 100 μL of the **DMAPy/BPO** nanoparticles were into the forepaws of live nude mice, followed by irradiating with 365 nm hand-held UV lamp (12 W) for 1 min and imaging with IVIS® instrument. During the imaging process, the mice were warmed with a heating pad under continued isoflurane anesthesia. The phosphorescent images were acquired in bioluminescence mode with open filter setting (exposure time: 17 s) and in fluorescence mode with Dsred filter setting (excitation: 430 nm, exposure time: auto).

10. Phosphorescent imaging of armpit tumor in vivo. 200 μL of **DMAPy/BPO** nanoparticles (4 mg mL-1 based on nanoparticles) were injected into the armpit tumor-bearing mice via the tail vein. At 6 h post-injection, the tumor bearing mice were irradiated with 365 nm hand-held UV lamp (12 W) for 1 min. During the imaging process, the mice were warmed with a heating pad under continued isoflurane anesthesia. The phosphorescent images were acquired in bioluminescence mode with open filter setting (exposure time: 17 s) and in fluorescence mode with Dsred filter setting (excitation: 430 nm, exposure time: auto). After that, the mice were sacrificed and main tissues including heart, lung, spleen, kidneys and liver were excised. These organs were imaged with the same conditions in fluorescence mode to obtain the phosphorescence signals.

11. Statistical analysis

The statistical analysis of normal hypothesis for the phosphorescence intensity of ions or tissues solution in each group was conducted using SPSS 21.0 statistical software (Lead Technologies, Chicago, USA). The phosphorescence intensity of ions or tissues solution in each group were expressed as mean ± standard deviation (SD). The differences between the groups were analyzed using a one-way analysis of variance (ANOVA), followed by Tukey's multiple comparisons test. Significant differences were defined as $p < 0.05$. (***).

Supplementary References

[1] Xu, S.; Wang, W.; Li, H.; Zhang, J.; Chen, R.; Wang, S.; Zheng, C.; Xing, G.; Song, C.; Huang, W. Design of highly efficient deep-blue organic afterglow through guest sensitization and matrices rigidification. *Nat. Commun.* **2020,** 11, 4802.

[2] Wei, P.; Zhang, X.; Liu, J.; Shan, G. G.; Zhang, H.; Qi, J.; Zhao, W.; Sung, H. H.; Williams, I. D.; Lam, J. W. Y.; Tang, B. Z. New Wine in Old Bottles: Prolonging room-temperature phosphorescence of crown ethers by supramolecular interactions. *Angew. Chem. Int. Ed.* **2020**, 59, 9293-9298.

[3] Wang, Y.; Yang, J.; Gong, Y.; Fang, M.; Li, Z.; Tang, B. Z. Host-guest materials with room

temperature phosphorescence: Tunable emission color and thermal printing patterns. *SmartMat* **2020**, 1, 1006.
[4] Zhang, Y.; Gao, L.; Zheng, X.; Wang, Z.; Yang, C.; Tang, H.; Qu, L.; Li, Y.; Zhao, Y. Ultraviolet irradiation-responsive dynamic ultralong organic phosphorescence in polymeric systems. *Nat. Commun.* **2021**, 12, 2297.
[5] Yang, J.; Gao, H.; Wang, Y.; Yu, Y.; Gong, Y.; Fang, M.; Ding, D.; Hu, W.; Tang, B. Z.; Li, Z. The odd–even effect of alkyl chain in organic room temperature phosphorescence luminogens and the corresponding in vivo imaging. *Mater. Chem. Front.* **2019**, 3, 1391-1397.
[6] Wang, Y.; Yang, J.; Tian, Y.; Fang, M.; Liao, Q.; Wang, L.; Hu, W.; Tang, B. Z.; Li, Z. Persistent organic room temperature phosphorescence: what is the role of molecular dimers? *Chem. Sci.* **2019**, 11, 833-838.
[7] Lei, Y.; Dai, W.; Tian, Y.; Yang, J.; Li, P.; Shi, J.; Tong, B.; Cai, Z.; Dong, Y. Revealing insight into long-lived room-temperature phosphorescence of host-guest systems. *J. Phys. Chem. Lett.* **2019**, 10, 6019-6025.
[8] Wang, J.; Chai, Z.; Wang, J.; Wang, C.; Han, M.; Liao, Q.; Huang, A.; Lin, P.; Li, C.; Li, Q.; Li, Z. Mechanoluminescence or room-temperature phosphorescence: molecular packing-dependent emission response. *Angew. Chem. Int. Ed.* **2019**, 58, 17297-17302.
[9] Shoji, Y.; Ikabata, Y.; Wang, Q.; Nemoto, D.; Sakamoto, A.; Tanaka, N.; Seino, J.; Nakai, H.; Fukushima, T. Unveiling a new aspect of simple arylboronic esters: Long-lived room-temperature phosphorescence from heavy-atom-free molecules. *J. Am. Chem. Soc.* **2017**, 139, 2728-2733.
[10] Alam, P.; Leung, N. L. C.; Liu, J.; Cheung, T. S.; Zhang, X.; He, Z.; Kwok, R. T. K.; Lam, J. W. Y.; Sung, H. H. Y.; Williams, I. D.; Chan, C. C. S.; Wong, K. S.; Peng, Q.; Tang, B. Z. Two are better than one: A design principle for ultralong-persistent luminescence of pure organics. *Adv. Mater.* **2020**, 32, 2001026.
[11] Gu, L.; Shi, H.; Bian, L.; Gu, M.; Ling, K.; Wang, X.; Ma, H.; Cai, S.; Ning, W.; Fu, L.; Wang, H.; Wang, S.; Gao, Y.; Yao, W.; Huo, F.; Tao, Y.; An, Z.; Liu, X.; Huang, W. Colour-tunable ultra-long organic phosphorescence of a single-component molecular crystal. *Nat. Photon.* **2019**, 13, 406-411.
[12] Ren, J.; Wang, Y.; Tian, Y.; Liu, Z.; Xiao, X.; Yang, J.; Fang, M.; Li, Z. Force-induced turn-on persistent room-temperature phosphorescence in purely organic luminogen. *Angew. Chem. Int. Ed.* **2021**, 60, 12335-12340.
[13] Tian, S.; Ma, H.; Wang, X.; Lv, A.; Shi, H.; Geng, Y.; Li, J.; Liang, F.; Su, Z. M.; An, Z.; Huang, W. Utilizing d-pπ bonds for ultralong organic phosphorescence. *Angew. Chem. Int. Ed.* **2019**, 58, 6645-6649.
[14] Liu, Z.-F.; Chen, X.; Jin, W. J. Ultralong lifetime room temperature phosphorescence and dual-band waveguide behavior of phosphoramidic acid oligomers. *J. Mater. Chem. C* **2020**, 8, 7330-7335.
[15] Li, M.; Ling, K.; Shi, H.; Gan, N.; Song, L.; Cai, S.; Cheng, Z.; Gu, L.; Wang, X.; Ma, C.; Gu, M.; Wu, Q.; Bian, L.; Liu, M.; An, Z.; Ma, H.; Huang, W. Prolonging ultralong organic phosphorescence lifetime to 2.5 s through confining rotation in molecular rotor. *Adv. Opt. Mater.* **2019**, 7, 1800820.
[16] Liu, X.; Dai, W.; Qian, J.; Lei, Y.; Liu, M.; Cai, Z.; Huang, X.; Wu, H.; Dong, Y. Pure room temperature phosphorescence emission of an organic host-guest doped system with a quantum efficiency of 64%. *J. Mater. Chem. C* **2021**, 9, 3391-3395.
[17] Nidhankar, A. D.; Goudappagouda; Mohana Kumari, D. S.; Chaubey, S. K.; Nayak, R.;

Gonnade, R. G.; Kumar, G. V. P.; Krishnan, R.; Babu, S. S. Self-assembled helical arrays for the stabilization of the triplet state. *Angew. Chem. Int. Ed.* **2020**, 59, 13079-13085.

[18] Jin, J.; Jiang, H.; Yang, Q.; Tang, L.; Tao, Y.; Li, Y.; Chen, R.; Zheng, C.; Fan, Q.; Zhang, K. Y.; Zhao, Q.; Huang, W. Thermally activated triplet exciton release for highly efficient tri-mode organic afterglow. *Nat. Commun.* **2020**, 11, 842.

[19] He, Z.; Gao, H.; Zhang, S.; Zheng, S.; Wang, Y.; Zhao, Z.; Ding, D.; Yang, B.; Zhang, Y.; Yuan, W. Z. Achieving persistent, efficient, and robust room-temperature phosphorescence from pure organics for versatile applications. *Adv. Mater.* **2019**, 31, 1807222.

[20] Cai, S.; Shi, H.; Li, J.; Gu, L.; Ni, Y.; Cheng, Z.; Wang, S.; Xiong, W. W.; Li, L.; An, Z.; Huang, W. Visible-light-excited ultralong organic phosphorescence by manipulating intermolecular interactions. *Adv. Mater.* **2017**, 29. 1701244.

[21] Xie, Y.; Ge, Y.; Peng, Q.; Li, C.; Li, Q.; Li, Z. How the molecular packing affects the room temperature phosphorescence in pure organic compounds: Ingenious molecular design, detailed crystal analysis, and rational theoretical calculations. *Adv. Mater.* **2017**, 29. 1606829.

[22] Wang, Y.; Yang, J.; Fang, M.; Yu, Y.; Zou, B.; Wang, L.; Tian, Y.; Cheng, J.; Tang, B. Z.; Li, Z. Förster resonance energy transfer: An efficient way to develop stimulus-responsive room-temperature phosphorescence materials and their applications. *Matter.* **2020**, 3, 449-463.

[23] Tang, L.; Zan, J.; Peng, H.; Yan, X.; Tao, Y.; Tian, D.; Yang, Q.; Li, H.; Chen, Q.; Huang, W.; Chen, R. X-ray excited ultralong room-temperature phosphorescence for organic afterglow scintillators. *Chem. Commun.* **2020**, 56, 13559-13562.

[24] Zhang, X.; Du, L.; Zhao, W.; Zhao, Z.; Xiong, Y.; He, X.; Gao, P. F.; Alam, P.; Wang, C.; Li, Z.; Leng, J.; Liu, J.; Zhou, C.; Lam, J. W. Y.; Phillips, D. L.; Zhang, G.; Tang, B. Z. Ultralong UV/mechano-excited room temperature phosphorescence from purely organic cluster excitons. *Nat. Commun.* **2019**, 10, 5161.

[25] Mao, Z.; Yang, Z.; Xu, C.; Xie, Z.; Jiang, L.; Gu, F. L.; Zhao, J.; Zhang, Y.; Aldred, M. P.; Chi, Z. Two-photon-excited ultralong organic room temperature phosphorescence by dual-channel triplet harvesting. *Chem. Sci.* **2019**, 10, 7352-7357.

[26] Gu, M.; Shi, H.; Ling, K.; Lv, A.; Huang, K.; Singh, M.; Wang, H.; Gu, L.; Yao, W.; An, Z.; Ma, H.; Huang, W. Polymorphism-dependent dynamic ultralong organic phosphorescence. *Research* **2020**, 2020, 8183450.

[27] Li, M.; Cai, X.; Qiao, Z.; Liu, K.; Xie, W.; Wang, L.; Zheng, N.; Su, S. J. Achieving high-efficiency purely organic room-temperature phosphorescence materials by boronic ester substitution of phenoxathiine. *Chem. Commun*. **2019**, 55, 7215-7218.

[28] Shi, H.; Song, L.; Ma, H.; Sun, C.; Huang, K.; Lv, A.; Ye, W.; Wang, H.; Cai, S.; Yao, W.; Zhang, Y.; Zheng, R.; An, Z.; Huang, W. Highly efficient ultralong organic phosphorescence through intramolecular-space heavy-atom effect. *J. Phys. Chem. Lett.* **2019**, 10, 595-600.

[29] Mao, Z.; Yang, Z.; Fan, Z.; Ubba, E.; Li, W.; Li, Y.; Zhao, J.; Yang, Z.; Aldred, M. P.; Chi, Z. The methylation effect in prolonging the pure organic room temperature phosphorescence lifetime. *Chem. Sci.* **2019**, 10, 179-184.

[30] Ishi, I. T.; Tanaka, H.; Park, I. S.; Yasuda, T.; Kato, S. I.; Ito, M.; Hiyoshi, H.; Matsumoto, T. White-light emission from a pyrimidine-carbazole conjugate with tunable phosphorescence-fluorescence dual emission and multicolor emission switching. *Chem. Commun.* 2020, 56, 4051-4054.

[31] Wang, J.; Gu, X.; Ma, H.; Peng, Q.; Huang, X.; Zheng, X.; Sung, S. H. P.; Shan, G.; Lam, J. W.

Y.; Shuai, Z.; Tang, B. Z. A facile strategy for realizing room temperature phosphorescence and single molecule white light emission. *Nat. Commun.* **2018**, 9, 2963.
[32] Yuan, J.; Wang, Y.; Li, L.; Wang, S.; Tang, X.; Wang, H.; Li, M.; Zheng, C.; Chen, R. Activating intersystem crossing and aggregation coupling by CN-substitution for efficient organic ultralong room temperature phosphorescence. *J. Phy. Chem. C* **2020**, 124, 10129-10134.
[33] Bi, X.; Shi, Y.; Peng, T.; Yue, S.; Wang, F.; Zheng, L.; Cao, Q. E. Multi-stimuli responsive and multicolor adjustable pure organic room temperature fluorescence-phosphorescent dual-emission materials. *Adv. Funct. Mater.* **2021**, 31, 2101312.
[34] Zhao, W.; Cheung, T. S.; Jiang, N.; Huang, W.; Lam, J. W. Y.; Zhang, X.; He, Z.; Tang, B. Z. Boosting the efficiency of organic persistent room-temperature phosphorescence by intramolecular triplet-triplet energy transfer. *Nat. Commun.* **2019**, 10, 1595.
[35] Zhang, L.; Li, M.; Gao, Q. Y.; Chen, C. F. An ultralong room-temperature phosphorescent material based on the combination of small singlet-triplet splitting energy and *H*-aggregation. *Chem. Commun.* **2020**, 56, 4296-4299.
[36] Sun, H.; Ding, R.; Lv, S.; Zhou, S.; Guo, S.; Qian, Z.; Feng, H. Clustering-triggered ultralong room-temperature phosphorescence of organic crystals through halogen-mediated molecular assembly. *J. Phys. Chem. Lett.* **2020**, 11, 4962-4969.
[37] Xu, L.; Li, G.; Xu, T.; Zhang, W.; Zhang, S.; Yin, S.; An, Z.; He, G. Chalcogen atom modulated persistent room-temperature phosphorescence through intramolecular electronic coupling. *Chem. Commun.* **2018**, 54, 9226-9229.
[38] Li, J. A.; Zhou, J.; Mao, Z.; Xie, Z.; Yang, Z.; Xu, B.; Liu, C.; Chen, X.; Ren, D.; Pan, H.; Shi, G.; Zhang, Y.; Chi, Z. Transient and persistent room-temperature mechanoluminescence from a white-light-emitting AIEgen with tricolor emission switching triggered by light. *Angew. Chem. Int. Ed.* **2018**, 57, 6449-6453.
[39] Xiao, F.; Wang, M.; Lei, Y.; Dai, W.; Zhou, Y.; Liu, M.; Gao, W.; Huang, X.; Wu, H. Achieving crystal-induced room temperature phosphorescence and reversible photochromic properties by strong intermolecular interactions. *J. Mater. Chem. C* **2020**, 8, 17410-17416.
[40] Feng, H. T.; Zeng, J.; Yin, P. A.; Wang, X. D.; Peng, Q.; Zhao, Z.; Lam, J. W. Y.; Tang, B. Z. Tuning molecular emission of organic emitters from fluorescence to phosphorescence through push-pull electronic effects. *Nat. Commun.* **2020**, 11, 2617.
[41] Tani, Y.; Komura, M.; Ogawa, T. Mechanoresponsive turn-on phosphorescence by a desymmetrization approach. *Chem. Commun.* **2020**, 56, 6810-6813.
[42] Wang, J. X.; Fang, Y. G.; Li, C. X.; Niu, L. Y.; Fang, W. H.; Cui, G.; Yang, Q. Z. Time-dependent afterglow color in a single-component organic molecular crystal. *Angew. Chem. Int. Ed.* **2020**, 59, 10032-10036.
[43] Chen, Y.; Xie, Y.; Shen, H.; Lei, Y.; Zhou, Y.; Dai, W.; Cai, Z.; Liu, M.; Huang, X.; Wu, H. Tunable phosphorescence/fluorescence dual emissions of organic isoquinoline-benzophenone doped systems by alkoxy engineering. *Chem. Eur. J.* **2020**, 26, 17376-17380.
[44] Chen, B.; Huang, W.; Su, H.; Miao, H.; Zhang, X.; Zhang, G. An unexpected chromophore-solvent reaction leads to bicomponent aggregation-induced phosphorescence. *Angew. Chem. Int. Ed.* **2020**, 59, 10023-10026.
[45] Wang, X.; Ma, H.; Gu, M.; Lin, C.; Gan, N.; Xie, Z.; Wang, H.; Bian, L.; Fu, L.; Cai, S.; Chi, Z.; Yao, W.; An, Z.; Shi, H.; Huang, W. Multicolor ultralong organic phosphorescence through alkyl engineering for 4D coding applications. *Chem. Mater*. **2019**, 31, 5584-5591.

[46] Yang, Z.; Mao, Z.; Zhang, X.; Ou, D.; Mu, Y.; Zhang, Y.; Zhao, C.; Liu, S.; Chi, Z.; Xu, J.; Wu, Y. C.; Lu, P. Y.; Lien, A.; Bryce, M. R. Intermolecular electronic coupling of organic units for efficient persistent room-temperature phosphorescence. *Angew. Chem. Int. Ed*. **2016**, 55, 2181-2185.
[47] Xu, Z.; Climent, C.; Brown, C. M.; Hean, D.; Bardeen, C. J.; Casanova, D.; Wolf, M. O. Controlling ultralong room temperature phosphorescence in organic compounds with sulfur oxidation state. *Chem. Sci.* **2021**, 12, 188-195.
[48] Salla, C. A. M.; Farias, G.; Rouzieres, M.; Dechambenoit, P.; Durola, F.; Bock, H.; de Souza, B.; Bechtold, I. H. Persistent solid-state phosphorescence and delayed fluorescence at room temperature by a twisted hydrocarbon. *Angew. Chem. Int. Ed.* **2019**, 58, 6982-6986.
[49] Xie, Z.; Zhang, X.; Wang, H.; Huang, C.; Sun, H.; Dong, M.; Ji, L.; An, Z.; Yu, T.; Huang, W. Wide-range lifetime-tunable and responsive ultralong organic phosphorescent multi-host/guest system. *Nat. Commun.* **2021**, 12, 3522.
[50] Cai, S.; Shi, H.; Tian, D.; Ma, H.; Cheng, Z.; Wu, Q.; Gu, M.; Huang, L.; An, Z.; Peng, Q.; Huang, W. Enhancing ultralong organic phosphorescence by effective π-type halogen bonding. *Adv. Funct. Mater.* **2018**, 28, 1705045.
[51] Liao, Q.; Gao, Q.; Wang, J.; Gong, Y.; Peng, Q.; Tian, Y.; Fan, Y.; Guo, H.; Ding, D.; Li, Q.; Li, Z. 9,9-Dimethylxanthene derivatives with room-temperature phosphorescence: substituent effects and emissive properties. *Angew. Chem. Int. Ed.* **2020**, 59, 9946-9951.
[52] Ma, X.; Jia, L.; Yang, B.; Li, J.; Huang, W.; Wu, D.; Wong, W.-Y. A color-tunable single molecule white light emitter with high luminescence efficiency and ultra-long room temperature phosphorescence. *J. Mater. Chem. C* **2021**, 9, 727-735.
[53] Wu, Z.; Nitsch, J.; Schuster, J.; Friedrich, A.; Edkins, K.; Loebnitz, M.; Dinkelbach, F.; Stepanenko, V.; Wurthner, F.; Marian, C. M.; Ji, L.; Marder, T. B. Persistent room temperature phosphorescence from triarylboranes: A combined experimental and theoretical study. *Angew. Chem. Int. Ed.* **2020**, 59, 17137-17144.
[54] Chen, X.; Liu, Z. F.; Jin, W. J. The effect of electron donation and intermolecular interactions on ultralong phosphorescence lifetime of 4-carnoyl phenylboronic acids. *J. Phys. Chem. A* **2020**, 124, 2746-2754.
[55] Xu, L.; Zhou, K.; Qiu, X.; Rao, B.; Pei, D.; Li, A.; An, Z.; He, G. Tunable ultralong organic phosphorescence modulated by main-group elements with different Lewis acidity and basicity. *J. Mater. Chem. C* **2020**, 8, 14740-14747.
[56] Wang, F.; Sun, J.; Liu, M.; Shi, H.; Ma, H.; Ye, W.; Wang, H.; Zhang, H.; An, Z.; Huang, W. D-A-D-type bipolar host materials with room temperature phosphorescence for high-efficiency green phosphorescent organic light-emitting diodes. *J. Mater. Chem. C* **2020**, 8, 1871-1878.
[57] Fateminia, S. M. A.; Mao, Z.; Xu, S.; Yang, Z.; Chi, Z.; Liu, B. Organic nanocrystals with bright red persistent room-temperature phosphorescence for biological applications. *Angew. Chem. Int. Ed.* **2017**, 56, 12160-12164.
[58] Wang, D.; Xie, Y.; Wu, X.; Lei, Y.; Zhou, Y.; Cai, Z.; Liu, M.; Wu, H.; Huang, X.; Dong, Y. Excitation-dependent triplet-singlet intensity from organic host-guest materials: Tunable color, white-light emission, and room-temperature phosphorescence. *J. Phys. Chem. Lett.* **2021**, 12, 1814-1821.
[59] Yu, Y.; Fan, Y.; Wang, C.; Wei, Y.; Liao, Q.; Li, Q.; Li, Z. Achieving enhanced ML or RTP performance: alkyl substituent effect on the fine-tuning of molecular packing. *Mater. Chem. Front.* **2021**, 5, 817-824.

[60] Tao, Y.; Chen, R.; Li, H.; Yuan, J.; Wan, Y.; Jiang, H.; Chen, C.; Si, Y.; Zheng, C.; Yang, B.; Xing, G.; Huang, W. Resonance-activated spin-flipping for efficient organic ultralong room-temperature phosphorescence. *Adv. Mater.* **2018**, 30, 1803856.
[61] Garain, S.; Kuila, S.; Garain, B. C.; Kataria, M.; Borah, A.; Pati, S. K.; George, S. J. Arylene diimide phosphors: aggregation modulated twin room temperature phosphorescence from pyromellitic diimides. *Angew. Chem. Int. Ed.* **2021**, 60, 12323-12327.
[62] Liu, Y.; Ma, Z.; Liu, J.; Chen, M.; Ma, Z.; Jia, X. Robust white-light emitting and multi-responsive luminescence of a dual-mode phosphorescence molecule. *Adv. Opt. Mater.* **2020**, 9, 2001685.
[63] Xiong, Y.; Zhao, Z.; Zhao, W.; Ma, H.; Peng, Q.; He, Z.; Zhang, X.; Chen, Y.; He, X.; Lam, J. W. Y.; Tang, B. Z. Designing efficient and ultralong pure organic room-temperature phosphorescent materials by structural isomerism. *Angew. Chem. Int. Ed.* **2018**, 57, 7997-8001.
[64] Li, H.; Li, H.; Wang, W.; Tao, Y.; Wang, S.; Yang, Q.; Jiang, Y.; Zheng, C.; Huang, W.; Chen, R. Stimuli-responsive crcularly polarized organic ultralong room temperature phosphorescence. *Angew. Chem. Int. Ed.* **2020**, 59, 4756-4762.
[65] Li, X. N.; Yang, M.; Chen, X. L.; Jia, J. H.; Zhao, W. W.; Wu, X. Y.; Wang, S. S.; Meng, L.; Lu, C. Z. Synergistic intra- and intermolecular noncovalent interactions for ultralong organic phosphorescence. *Small* **2019**, 15, 1903270.
[66] Yuan, J.; Chen, R.; Tang, X.; Tao, Y.; Xu, S.; Jin, L.; Chen, C.; Zhou, X.; Zheng, C.; Huang, W. Direct population of triplet excited states through singlet-triplet transition for visible-light excitable organic afterglow. *Chem. Sci.* **2019**, 10, 5031-5038.
[67] Yuan, J.; Wang, S.; Ji, Y.; Chen, R.; Zhu, Q.; Wang, Y.; Zheng, C.; Tao, Y.; Fan, Q.; Huang, W. Invoking ultralong room temperature phosphorescence of purely organic compounds through *H*-aggregation engineering. *Mater. Horiz.* **2019**, 6, 1259-1264.
[68] He, Z.; Zhao, W.; Lam, J. W. Y.; Peng, Q.; Ma, H.; Liang, G.; Shuai, Z.; Tang, B. Z. White light emission from a single organic molecule with dual phosphorescence at room temperature. *Nat. Commun.* **2017**, 8, 416.
[69] Bhattacharjee, I.; Acharya, N.; Karmakar, S.; Ray, D. Room-temperature orange-red phosphorescence by way of intermolecular charge transfer in single-component phenoxazine-quinoline conjugates and chemical sensing. *J. Phys. Chem. C* **2018**, 122, 21589-21597.
[70] Shi, H.; Zou, L.; Huang, K.; Wang, H.; Sun, C.; Wang, S.; Ma, H.; He, Y.; Wang, J.; Yu, H.; Yao, W.; An, Z.; Zhao, Q.; Huang, W. A highly efficient red metal-free organic phosphor for time-resolved luminescence imaging and photodynamic therapy. *ACS Appl. Mater. Interfaces* **2019**, 11, 18103-18110.
[71] Chen, X.; Xu C.; Wang, T.; Zhou, Cao.; Du, J.; Wang, Z.; Xu, H.; Xie, T.; Bi, G.; Jiang, J.; Zhang, X.; Demas, J. N.; Trindle, Carl. O.; Luo, Y.; Zhang, G. Q. Versatile room-temperature-phosphorescent materials prepared from *N*-substituted naphthalimides: emission enhancement and chemical conjugation. *Angew. Chem. Int. Ed.* **2016**, 55, 9872-9876.
[72] Dou, X.; Zhu, T.; Wang, Z.; Sun, W.; Lai, Y.; Sui, K.; Tan, Y.; Zhang, Y.; Yuan, W. Z. Color-tunable, excitation-dependent, and tme-dependent afterglows from pure organic amorphous polymers. *Adv. Mater.* **2020**, 32, 2004768.
[73] Xiao, L.; Wu, Y.; Yu, Z.; Xu, Z.; Li, J.; Liu, Y.; Yao, J.; Fu, H. Room-temperature phosphorescence in pure organic materials: halogen bonding switching effects. *Chem. Eur. J.* **2018**, 24, 1801-1805.

[74] Huang, L.; Liu, L.; Li, X.; Hu, H.; Chen, M.; Yang, Q.; Ma, Z.; Jia, X. Crystal-state photochromism and dual-mode mechanochromism of an organic molecule with fluorescence, room-temperature phosphorescence, and delayed fluorescence. *Angew. Chem. Int. Ed.* **2019**, 58, 16445-16450.
[75] Tu, D.; Cai, S.; Fernandez, C.; Ma, H.; Wang, X.; Wang, H.; Ma, C.; Yan, H.; Lu, C.; An, Z. Boron-cluster-enhanced ultralong organic phosphorescence. *Angew. Chem. Int. Ed.* **2019**, 58, 9129-9133.
[76] He, G.; Du, L.; Gong, Y.; Liu, Y.; Yu, C.; Wei, C.; Yuan, W. Z. Crystallization-induced red phosphorescence and grinding-induced blue-shifted emission of a benzobis(1,2,5-thiadiazole)-thiophene conjugate. *ACS Omega* **2019**, 4, 344-351.
[77] Wang, X. F.; Guo, W. J.; Xiao, H.; Yang, Q. Z.; Chen, B.; Chen, Y. Z.; Tung, C. H.; Wu, L. Z. Pure organic room temperature phosphorescence from unique micelle-assisted assembly of nanocrystals in water. *Adv. Funct. Mater.* **2020**, 30, 1907282.
[78] An, Z.; Zheng, C.; Tao, Y.; Chen, R.; Shi, H.; Chen, T.; Wang, Z.; Li, H.; Deng, R.; Liu, X.; Huang, W. Stabilizing triplet excited states for ultralong organic phosphorescence. *Nat. Mater.* **2015**, 14, 685-690.
[79] Wang, X. F.; Xiao, H.; Chen, P. Z.; Yang, Q. Z.; Chen, B.; Tung, C. H.; Chen, Y. Z.; Wu, L. Z. Pure organic room temperature phosphorescence from excited dimers in self-assembled nanoparticles under visible and near-infrared irradiation in water. *J. Am. Chem. Soc.* **2019**, 141, 5045-5050.
[80] Wu, H.; Zhao, P.; Li, X.; Chen, W.; Agren, H.; Zhang, Q.; Zhu, L. Tuning for visible fluorescence and near-infrared phosphorescence on a unimolecular mechanically sensitive platform via adjustable CH-π interaction. *ACS Appl. Mater. Interfaces* **2017**, 9, 3865-3872.
[81] Su, Y.; Zhang, Y.; Wang, Z.; Gao, W.; Jia, P.; Zhang, D.; Yang, C.; Li, Y.; Zhao, Y. Excitation-dependent long-life luminescent polymeric systems under ambient conditions. *Angew. Chem. Int. Ed.* **2020**, 59, 9967-9971.
[82] Gutierrez, G. D.; Sazama, G. T.; Wu, T.; Baldo, M. A.; Swager, T. M. Red phosphorescence from benzo[2,1,3]thiadiazoles at room temperature. *J. Org. Chem.* **2016**, 81, 4789-4796.
[83] Wang, J.-X.; Zhang, H.; Niu, L.-Y.; Zhu, X.; Kang, Y.-F.; Boulatov, R.; Yang, Q.-Z. Organic composite crystal with persistent room-temperature luminescence above 650 nm by combining triplet-triplet energy transfer with thermally activated delayed fluorescence. *CCS Chem.* **2020**, 2, 1391-1398.
[84] Kuila, S.; Ghorai, A.; Samanta, P. K.; Siram, R. B. K.; Pati, S. K.; Narayan, K. S.; George, S. J. Red-emitting delayed fluorescence and room temperature phosphorescence from core-Substituted naphthalene diimides. *Chem. Eur. J.* **2019**, 25, 16007-16011.
[85] Wu, X.; Huang, C. Y.; Chen, D. G.; Liu, D.; Wu, C.; Chou, K. J.; Zhang, B.; Wang, Y.; Liu, Y.; Li, E. Y.; Zhu, W.; Chou, P. T. Exploiting racemism enhanced organic room-temperature phosphorescence to demonstrate Wallach's rule in the lighting chiral chromophores. *Nat. Commun.* **2020**, 11, 2145.
[86] Ono, T.; Kimura, K.; Ihara, M.; Yamanaka, Y.; Sasaki, M.; Mori, H.; Hisaeda, Y. Room-temperature phosphorescence emitters exhibiting red to near-infrared emission derived from intermolecular charge-transfer triplet states of naphthalenediimide-halobenzoate triad molecules. *Chem. Eur. J.* **2021**. DOI:10.1002/chem.202100906.
[87] Dang, Q.; Jiang, Y.; Wang, J.; Wang, J.; Zhang, Q.; Zhang, M.; Luo, S.; Xie, Y.; Pu, K.; Li, Q.;

Li, Z. Room-temperature phosphorescence resonance energy transfer for construction of near-infrared afterglow imaging agents. *Adv. Mater.* **2020**, 32, 2006752.
[88] Sun, S.; Ma, L.; Wang, J.; Ma, X.; Tian, H. Red-light excited efficient metal-free near-infrared room-temperature phosphorescent films. *Natl. Sci. Rev.* **2021**, DOI:10.1093/nsr/nwab085.
[89] Liao, F.; Du, J.; Nie, X.; Wu, Z.; Su, H.; Huang, W.; Wang, T.; Chen, B.; Jiang, J.; Zhang, X.; Zhang, G. Modulation of red organic room-temperature phosphorescence in heavy atom-free phosphors. *Dyes. Pigm.* **2021**, 193, 109505.
[90] Paisley, N. R.; Halldorson, S. V.; Tran, M. V.; Gupta, R. M.; Kamal, S.; Algar, W. R.; Hudson, Z. M. Near-infrared emitting boron difluoride curcuminoid-based polymers exhibiting thermally activated delayed fluorescence as biological imaging probes. *Angew. Chem. Int. Ed.* **2021**, 60,18630-18638.
[91] Zhu, T. W.; Yang, T. J.; Zhang, Q.; Yuan, W. Z. Clustering and Halogen Effects Enabled Red/Near-Infrared Room Temperature Phosphorescence from Aliphatic Cyclic Imides. *ChemRxiv*. **2021**., DOI: 10.26434/chemrxiv.14174240.v1.
